# Bacterial protein acetylation: mechanisms, functions, and methods for study

**DOI:** 10.3389/fcimb.2024.1408947

**Published:** 2024-07-04

**Authors:** Jocelin Rizo, Sergio Encarnación-Guevara

**Affiliations:** Laboratorio de Proteómica, Centro de Ciencias Genómicas, Universidad Nacional Autónoma de México, Cuernavaca, Mexico

**Keywords:** bacteria, protein, acetylation, functions, methods

## Abstract

Lysine acetylation is an evolutionarily conserved protein modification that changes protein functions and plays an essential role in many cellular processes, such as central metabolism, transcriptional regulation, chemotaxis, and pathogen virulence. It can alter DNA binding, enzymatic activity, protein-protein interactions, protein stability, or protein localization. In prokaryotes, lysine acetylation occurs non-enzymatically and by the action of lysine acetyltransferases (KAT). In enzymatic acetylation, KAT transfers the acetyl group from acetyl-CoA (AcCoA) to the lysine side chain. In contrast, acetyl phosphate (AcP) is the acetyl donor of chemical acetylation. Regardless of the acetylation type, the removal of acetyl groups from acetyl lysines occurs only enzymatically by lysine deacetylases (KDAC). KATs are grouped into three main superfamilies based on their catalytic domain sequences and biochemical characteristics of catalysis. Specifically, members of the GNAT are found in eukaryotes and prokaryotes and have a core structural domain architecture. These enzymes can acetylate small molecules, metabolites, peptides, and proteins. This review presents current knowledge of acetylation mechanisms and functional implications in bacterial metabolism, pathogenicity, stress response, translation, and the emerging topic of protein acetylation in the gut microbiome. Additionally, the methods used to elucidate the biological significance of acetylation in bacteria, such as relative quantification and stoichiometry quantification, and the genetic code expansion tool (CGE), are reviewed.

## 1 Introduction

Post-translational modifications (PTMs) are covalent and generally enzymatic modifications of amino acid residues in a protein that occur after it is synthesized. Amino acid side chains may be modified by adding small chemical groups (e.g., methylation, acetylation, and phosphorylation) or more complex molecules such as oligosaccharides or small peptides (e.g., glycosylation and ubiquitylation) (Mann and Jensen, 2003; Heuts et al., 2009; Macek et al., 2019). The PTMs most widely distributed and frequently reported are phosphorylation, glycosylation, ubiquitination, methylation, and acetylation, among other alkylations (Khoury et al., 2011). Multiple PTMs can occur on a single type of amino acid, or different types of amino acids may be given the same modification, thereby altering the chemical properties of the modified sites. Through protein PTMs, cells regulate their functions and metabolic pathways and increase the variety and complexity of target proteins (Shahin and Javad, 2021).

A wide variety of amino acid residues can be post-translationally acetylated, including:

(1) The irreversible acetylation of the alpha-amino group in the N-terminal amino acid of proteins (Nα-acetylation), a common modification in eukaryotes (50–70% of yeast proteins and 80–90% of human proteins), but rare in prokaryotes.
(2) The reversible acetylation of the hydroxyl side chain of serine or threonine (O-acetylation) is detected only in a few eukaryotic organisms.
(3) The acetylation of serine, threonine, and histidine by a family of bacterial effector molecules from some pathogenic species (Mukherjee et al., 2007; Mittal et al., 2010; Paquette et al., 2012; Christensen et al., 2019b).
(4) The reversible acetylation of the epsilon-amino group of the lysine residue (Nε-acetylation), a modification found in eukaryotes and prokaryotes (Khoury et al., 2011; Diallo et al., 2019; Ramazi and Zahiri, 2021). The lysine epsilon amino group is also the site of other modifications besides acetylation (Gil et al., 2017).

**Figure 1:**
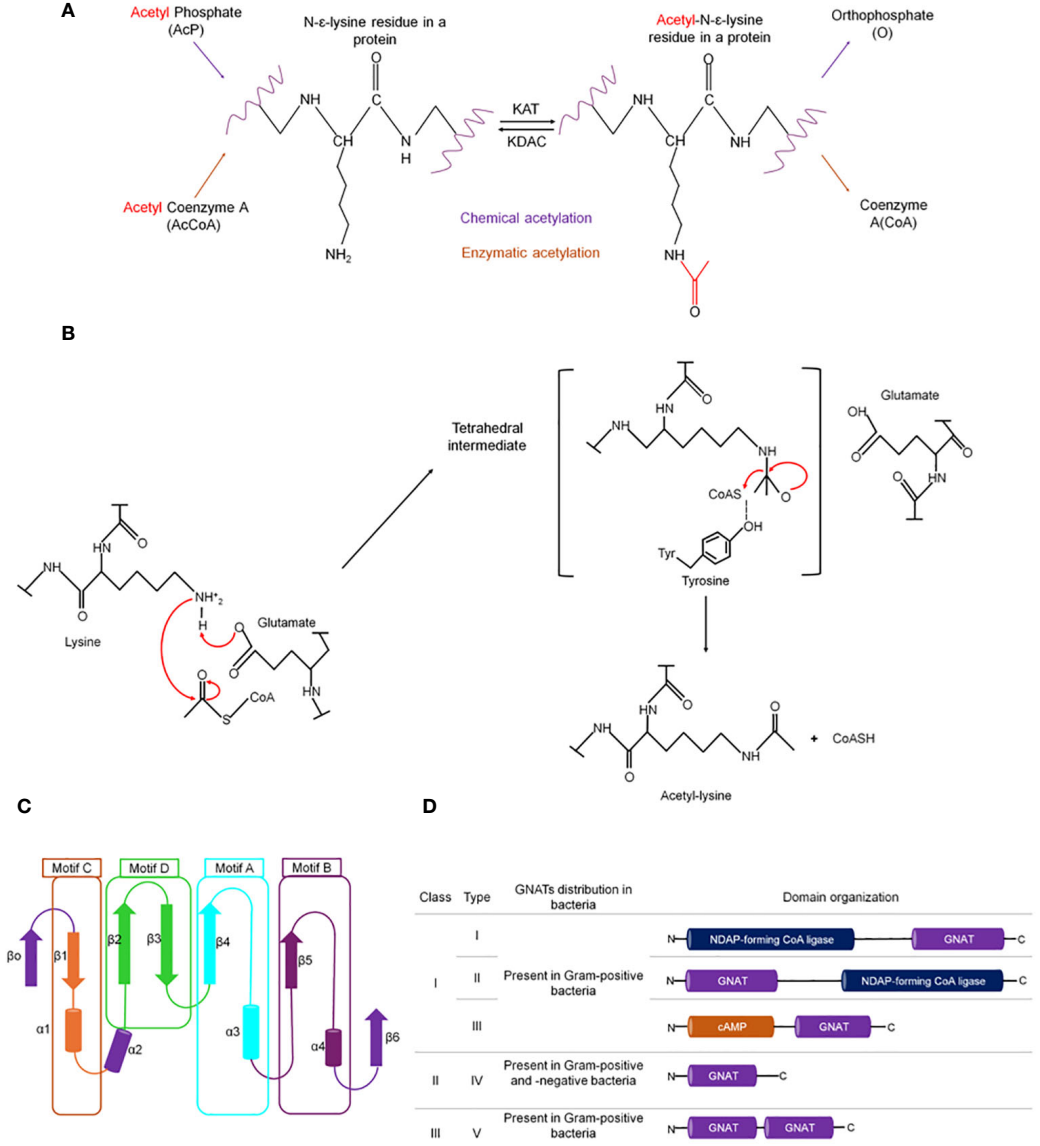
Types of lysine acetylation in prokaryotes. **(A)** Enzymatic and chemical acetylation and their respective acetyl donor molecule. In bacteria, enzymatic acetylation is catalyzed by Gcn5-related N-acetyltransferase (GNAT). **(B)** The catalytic mechanism exerted by GNATs is a sequential mechanism where glutamate acts as a general base to deprotonate the amino group of the lysine, enabling nucleophilic attack of the acetyl-CoA carbonyl, followed by the formation of a transient tetrahedral intermediate, that is resolved to yield the acetylated substrate amino group and coenzyme **(A)** Overall structure of GNATs: **(C)** Cartoon representation of GNATS topology. The secondary structure elements are colored and represent the different motifs. Motifs C (β1–α1), D (β2–β3), A (β4–α3), and B (β5–α4) are colored orange, green, aquamarine, and blue, respectively. The least conserved secondary structure elements (strands β0 and β6 and helix α2) absent in some GNAT proteins are colored purple. **(D)** Classification of GNATs in prokaryotes based on their domain organization and the arrangement of the GNAT domain [figure redrawn and modified from VanDrisse and Escalante-Semerena, 2019; Blasl et al., 2021; Lammers, 2021].

Nε-acetylation occurs either enzymatically by the lysine acetyltransferases (KATs) or non-enzymatically by chemical acetylation. In both cases, the acetyl group comes from a donor molecule, typically acetyl coenzyme A (AcCoA) or acetyl phosphate (AcP). Both types of acetylation are reversible by the action of deacetylase enzymes (**Figure 1A**) (Hentchel and Escalante-Semerena, 2015).

Lysine acetylation was discovered 60 years ago in histones and was implicated in transcriptional regulation (Allfrey et al., 1964). Extensive studies in eukaryotic cells have shown that acetylation is an essential protein modification that influences many cellular processes, including protein-protein interaction, protein stability, protein folding, cellular localization, and enzymatic activity. This PTM regulates different biological pathways, such as cell cycle control, cell metabolism, DNA repair, DNA replication, ribosome biogenesis in the nucleus, nuclear transport, translation, and transcription, among others (Berrabah et al., 2011; Oliveira and Sauer, 2012; Gil et al., 2017; Tarazona and Pourquié, 2020). In contrast, research on acetylation in bacteria is relatively new and primarily focused on describing global acetylation through a proteomic approach. The first studies on acetylation as a regulatory mechanism were carried out in *Salmonella enterica*, where it was demonstrated that the acetyl-CoA synthetase (Acs), an enzyme responsible for the synthesis of acetyl-CoA (Ac-CoA) from acetate, is post-translationally regulated by the protein acetyltransferase (Pat) that acetylates Lys residue 609 of Acs (Starai et al., 2002, Starai et al., 2004). Furthermore, it was found that Acs is activated by CobB, a bacterial protein deacetylase homologous to yeast Sir2, which specifically hydrolyzes the acetyl group of the acetyl-lysine active site of Acs to promote its activity (Starai et al., 2002, Starai et al., 2004). Since then, acetylation in bacteria has been shown to regulate several fundamental pathways, including cellular function, physiology, and metabolism. Proteomics studies have found that in some bacteria, the proteins acetylated on lysine constitute more than 10% of the proteome.

## 2 Enzymatic acetylation

Nε-acetylation refers to adding an acetyl group from a donor molecule, like AcCoA, to an epsilon amino group of lysine side chains. The modification increases the size of the side chain and neutralizes the positive charge of the unmodified amino acid. Nε-acetylation is a reversible process where the acetate added by KATs can be removed by histone deacetylases (HDAC) (Taunton et al., 1996; Haigis and Guarente, 2006; Yang and Seto, 2008; Bürger and Chory, 2018; VanDrisse and Escalante-Semerena, 2019). These opposing enzymatic activities determine the levels of posttranslational modification on a particular protein.

KATs are grouped into three major families based on amino acid sequence homology and biochemical characteristics of catalysis: (i) Gcn5- related *N*-acetyltransferases (GNATs), (ii) the p300/CBP family, and (iii) MYST family. The MYST and p300/CBP families are present only in eukaryotic cells, while Gcn5-related *N*-acetyltransferase family members occur in bacteria, eukaryotes, and archaea (Gu and Roeder, 1997; Vetting et al., 2005; Lee and Workman, 2007; Finkel et al., 2009; Favrot et al., 2016).

KAT families differ in sequence similarity, domain organization, substrate specificity, and catalytic mechanism. The GNAT family uses two main types of kinetic mechanisms: a sequential mechanism and a ping-pong mechanism. In the direct transfer mechanism, AcCoA and the protein substrate bind to form a ternary complex in which active site glutamate or aspartate acts as a general base to deprotonate the amino group of the lysine, allowing it to perform a nucleophilic attack on the thioester carbonyl carbon of the acetyl moiety of AcCoA (**Figure 1B**) (Liu et al., 2008). In the ping-pong mechanism, the acetyl group is covalently attached to the enzyme to form an acyl-enzyme intermediate before being transferred to the substrate. For this type of mechanism, the presence of nucleophilic residues such as cysteine or serine in a proper position in the active site is required (Vetting et al., 2005; Majorek et al., 2013; Baumgartner et al., 2021). Although the MYST family members can also use the sequential or ping-pong mechanisms, this catalysis route occurs via an acetyl-cysteine intermediate (Yan et al., 2002). In contrast, the p300/CBP family does not use a catalytic base to initiate the transfer of the acyl moiety. Instead, this family uses a Theorell–Chance mechanism where a tyrosine in the active site acts as a catalytic acid to increase the nucleophilicity of the lysine side chain (Liu et al., 2008). The protein substrate-AcCoA association binds transiently to the enzyme surface, allowing the lysine residue to receive the acetyl group, followed by rapid protein dissociation (Zhang et al., 2014). The ternary complex formed is kinetically irrelevant for catalysis (Liu et al., 2008).

### 2.1 GNAT family in prokaryotes

As mentioned, members of the GNATs family are found in all domains of life and are involved in diverse processes such as synthesis, transcription control, antibiotic resistance, stress regulation, metabolic flux regulation, and virulence regulation (Xie et al., 2014; Hentchel and Escalante-Semerena, 2015; Favrot et al., 2016). The first two reported GNAT members were the aminoglycoside *N*-acetyltransferase from multidrug-resistant *Serratia marcescens* and the histone acetyltransferase (HAT1) from *Saccharomyces cerevisiae* (Dutnall et al., 1998; Wolf et al., 1998). So far, GNATs comprise one of the largest superfamilies, with more than 300,000 GNAT proteins identified (Salah Ud-Din et al., 2016; Burckhardt and Escalante-Semerena, 2020; Krtenic et al., 2020).

GNATs can acetylate a wide variety of substrates, from antibiotics to proteins and peptides (**Table 1**), so they constitute a highly diverse family of proteins. Interestingly, some acetyltransferases can acetylate multiple substrates. For example, the Eis protein from *Mycobacterium tuberculosis* was initially described as an aminoglycoside acetyltransferase (Zaunbrecher et al., 2009; Chen et al., 2011; Houghton et al., 2013), but it also acetylates proteins (Kim et al., 2012; Ghosh et al., 2016). A deeper analysis of the GNAT superfamily members’ functions and their target molecules have been discussed in several reviews (Dyda et al., 2000; Vetting et al., 2005; Favrot et al., 2016; Salah Ud-Din et al., 2016; VanDrisse and Escalante-Semerena, 2019; Burckhardt and Escalante-Semerena, 2020).

**Table 1:** Some examples of GNAT enzymes, their targets, and the effect produced by the acetylation are shown.

Family	Name	Bacteria	Substrate	Effect	Reference
Aminoglycoside N-acetyltransferase	MtAAC(2’)-Ic	*M. tuberculosis*	Aminoglycosides	Antibiotic resistance	Barrett et al., 2008
	EfAAC(6’)-Ii	*Enterococcus faecium*			Wright and Ladak, 1997; Magnet et al., 2001; Hegde et al., 2002
	SeAAC(6’)Ie	*Salmonella enterica*			
	MtbEis	*M. tuberculosis*	Aminoglycosides	Antibiotic resistance	Zaunbrecher et al., 2009; Chen et al., 2011; Houghton et al., 2013;
Spermidine/spermine N-acetyltransferase	BltD	*B. subtilis*	Polyamines (spermidine /spermine)	Unknown	Woolridge et al., 1999
	PaiA	*B. subtilis*			Forouhar et al., 2005
	SpeG	*Vibrio cholerae*		Possibly involved in biofilm formation	Filippova et al., 2015
		*Staphylococcus aureus*			Li et al., 2019
		*E. coli*		Inhibition of spermidine accumulation	Fukuchi et al., 1995; Limsuwun and Jones, 2000
		*Bacillus thuringiensis*		Unknown	Tsimbalyuk et al., 2020
	Ste26	*Streptomyces* sp.		Unknown	Bai et al., 2011
	PmvE	*Enterococcus faecalis*	Putrescine/spermine	Contributes to the virulence of the bacteria. Possibly involved in biofilm formation	Martini et al., 2015
Lysine Nϵ-acyltransferase	MtRv1347c	*M. tuberculosis*	Fatty acyl-CoA with longer chain lengths	Possible role in siderophore biosynthesis (mycobactin T)	Card et al., 2005; Christensen et al., 2019b; Frankel and Blanchard, 2008; Hentchel and Escalante-Semerena, 2015;

Non-histone protein N-acetyltransferases	YfiQ and its homologs	*E. coli, Vibrio cholerae, Salmonella entérica, Yersinia pestis, Rhodopseudomonas palustris, Streptomyces lividans*, and *Streptomyces* *griseus*	Acetyl-CoA synthetase	Loss of enzyme activity	Starai et al., 2004
	MSMEG_5458	*M. tuberculosis*			Xu et al., 2011
	PA4794	*P. aeruginosa*	C-terminal lysine residue of a peptide	No reported	Majorek et al., 2013
	MtbEis	*M. tuberculosis*	Nucleoid-associated protein (MtHU)	May cause reduced interaction with DNA and altered DNA compaction ability of nucleoid-associated proteins	Ghosh et al., 2016
			Dual-specificity protein phosphatase 16/mitogen-activated protein kinase phosphatase-7 (DUSP16/MKP-7)	Inhibition of JNK-dependent autophagy, phagosome maturation, and ROS generation	Kim et al., 2012

### 2.2 Overall structure of GNATs

Although the primary sequence similarity between GNATs is moderate (3%–23%), they are folded into a highly conserved three-dimensional structure. The core secondary elements of the GNAT proteins consist of six or seven β-strands and four α-helices, connected in the order β0-β1-α1-α2-β2-β3-β4-α3-β5-α4-β6. Four conserved motifs, known as the *N*-acetyltransferase domain, are found in this core arranged in the order C-D-A-B (**Figure 1C**) (Tercero et al., 1992; Dyda et al., 2000; Hentchel and Escalante-Semerena, 2015).

Motifs C and D are essential in protein stability, while motifs A and B contain the residues involved in acyl-CoA and acceptor substrate binding, respectively (Dyda et al., 2000; Vetting et al., 2005). Motif A contains a six-residue segment (Q/R)-X-X-G-X-(G/A) known as the P-loop (phosphate-binding loop), that serves for substrate recognition and binding and contains glutamic or aspartic acid residues to deprotonate the target lysine (Vetting et al., 2005; Liu et al., 2008; Lu et al., 2017). Across the entire GNAT superfamily, there is structural divergence in the β1β2 loop, the α-4 helix of motif B, and strand β6 at the C-terminal, which together form the binding site for the acceptor substrate. Specifically, the C-terminal region contains a loop of varying length and position, and the residues in the motif B are not well conserved. These structural variants recognize different substrates (Dyda et al., 2000; Salah Ud-Din et al., 2016). For example, in the mycothiol synthase (Rv0819) from *M. tuberculosis*, the β-strand 1 in the N-terminal domain is missing, the position of helix 2, and a long loop inserted between α3′ and β5′, while aminoglycoside 6´-N-acetyltransferase from *Enterococcus faecium* has an additional α-helix between the β1 and β2 strands (Wybenga-Groot et al., 1999; Vetting et al., 2003).

The binding of the enzyme to AcCoA/CoA occurs through interactions with the pantetheine and pyrophosphate moieties of CoA (Dutnall et al., 1998; Clements et al., 1999; Lin et al., 1999; Rojas et al., 1999; Wang et al., 2008; Hentchel and Escalante-Semerena, 2015). The pantetheine binding is based on hydrogen bonds with the main chain of β4 and β 5, and the pyrophosphate is coordinated mainly by the main chain nitrogen atoms of the conserved phosphate binding loop between β4 and α3 (Majorek et al., 2017).

### 2.3 GNAT classification

Different works have proposed a classification system for bacterial KATs of the GNAT family (**Figure 1D**) (Hentchel and Escalante-Semerena, 2015; Lu et al., 2017; Christensen et al., 2019a; VanDrisse and Escalante-Semerena, 2019). This classification system recognized that the GNAT family exhibits different sequence lengths, domain architecture, and types. Recently, Christensen et al., 2019a proposed a new system of three main classes of KATs based on sequence length and the present GNAT domains, as well as five different types of KATs based on domain identities and arrangements. Class I consists of a large (>80 KDa) multidomain enzyme, where only the GNAT catalytic domain is conserved. Class II encompasses most bacterial acetyltransferases, smaller enzymes with a single GNAT domain. Class III has a dual arrangement of GNAT domains. Depending on domain position, these classes are further categorized into five types: types I and II contain a domain homologous to nucleotide-diphosphate (NDP)-forming acyl-CoA ligase/synthetase (700–900 aa) but which lack activity and a GNAT domain (~200 aa) at the N- or C-terminal. Type III KATs have a smaller regulatory domain (~300–400 aa) at the N-terminal that binds to an effector (e.g., cAMP, NADP, or amino acids) and a C-terminal GNAT domain. Type IV and V do not contain any regulatory domain and consist only of one GNAT domain (400 aa) and multiple GNAT domains, respectively (**Figure 1D**) (Hentchel and Escalante-Semerena, 2015; Lu et al., 2017; Christensen et al., 2019a; VanDrisse and Escalante-Semerena, 2019).

The diversity of domain architectures and organization of GNATs indicate that in bacteria, lysine acetylation is regulated by diverse metabolic signals in response to physiological conditions and environmental changes. However, more studies are needed to elucidate the substrates and structures of these enzymes and their role in these organisms.

## 3 Non-enzymatic acetylation

Acetyl-CoA is synthesized by the oxidative decarboxylation of pyruvate during glycolysis, the catabolism of isoleucine, leucine, and threonine, the reverse TCA cycle, and fatty acid β-oxidation. Whereas KATs use AcCoA to acetylate proteins, it can also modify the amino group of the lysine side chains without the assistance of an enzyme. Non-enzymatic acetylation (chemical acetylation) was first mentioned for histone proteins by Phillips, 1963. A few years later, it was demonstrated that purified histones, albumin, and synthetic lysine homopolymer are acetylated *in vitro* at a pH ~9 in the presence of AcCoA without enzymes (Paik et al., 1970). In eukaryotic cells, specifically in the mitochondria, the absence of acetyltransferases and the unique conditions, such as the high concentration of AcCoA (1.5 mM steady-state) and alkaline pH (7.9–8.0), suggests that lysine acetylation occurs chemically. For example, many mitochondrial proteins are acetylated under conditions mimicking those of the mitochondria matrix in a mechanism that is generally pH- and acyl-CoA concentration-dependent (Wagner and Payne, 2013).

Direct chemical acetylation has also been observed in bacteria. Both AcCoA and AcP can non-enzymatically acetylate lysine residues on many proteins. On the one hand, demonstrating that non-enzymatic AcCoA-dependent acetylation occurs *in vivo* is difficult since AcCoA is an essential molecule, and deleting it would result in a lack of viability of the organisms. However, it has been shown that AcCoA can acetylate purified bacterial proteins at a concentration comparable to or below physiological levels (Barak et al., 2006; Ramos-Montañez et al., 2010; Sun et al., 2016; Wang et al., 2017). On the other hand, the reactive chemical properties of AcP support the hypothesis that it could function directly as an acetyl donor. In water, AcP hydrolyzes quickly under basic or acidic conditions in the presence of Mg2+ or Ca2+ (Lipmann and Tuttle, 1944; Koshland and Daniel, 1952). Also, acyl group transfer is favored over hydrolysis in the presence of nucleophilic reagents such as thiols, hydroxyl groups, or the amino groups of lysine side chains (Jencks, 1969; Kuhn et al., 2014).

In bacteria, substantial evidence has shown that AcP-dependent non-enzymatic acetylation directly correlates with the levels of this high-energy intermediate. When cells are unable to produce AcP, acetylation levels are reduced, whereas when cells accumulate it, the acetylation levels increase significantly (Weinert et al., 2013; Kuhn et al., 2014; Kosono et al., 2015; Post et al., 2017; Bontemps-Gallo et al., 2018). This PTM seems more global and less specific than enzymatic acetylation (Weinert et al., 2013).

### 3.1 AcP is the donor molecule in bacteria chemical acetylation

Many studies on protein acetylation in bacteria have focused on describing enzymatic acetylation and its implication in cellular physiology, pathogenesis, and response to environmental conditions. However, non-enzymatic acetylation is also possible (**Table 2**). The acetylation by AcP has been mainly described by proteomic studies, in which a comparative analysis of the acetylome of different strains allows us to determine if there is any difference in the protein acetylation levels and infer the acetylation mechanism. This has been achieved in *Escherichia coli, Neisseria gonorrhoeae* and *Borrelia burgdorferi* (**Table 2**) (Weinert et al., 2013; Kuhn et al., 2014; Kosono et al., 2015; Schilling et al., 2015; Post et al., 2017; Bontemps-Gallo et al., 2018; Reverdy et al., 2018). These studies have focused on comparing the proteomic data of the acetylated proteins of the wild-type strain with different isogenic mutant strains. The acetylome data using *E. coli* as a model demonstrated that acetylation depends on AcP formation and occurs at a low level in growth-arrested cells. Mutant cells unable to synthesize (*pta ackA* mutant) or metabolize (*ackA* mutant) AcP had the opposite behavior, with the former mutant having significantly reduced acetylation levels and the latter mutant, which accumulated AcP, having substantially higher acetylation levels. It was also shown that AcP acetylates lysine residues *in vitro* at a concentration comparable to that found *in vivo*. These data establish AcP as a critical donor for acetylation and suggest that AcP acts nonenzymatically to regulate acetylation levels in response to glucose (**Table 2**) (Weinert et al., 2013; Kuhn et al., 2014; Schilling et al., 2015).

**Table 2:** Acetylome studies show that AcP is the primary source of acetylation.

		Tested condition				
Bacteria	Mutant	Time/growth phase	Medium	Lysine acetylation sites	Acetylated proteins	Reference
*E. coli*	Wild-type	EP	Not mentioned			Weinert et al., 2013
	ackA			Increase of 8–8-fold of global acetylation		
	pta			Reduce by about 40% of global acetylation		
	Wild-type	SP	TB7/ TB7+glucose	780/ 1204	355/ 446	Kuhn et al., 2014
	ackA			1149/ 2473	448/ 751	
	*pta ackA*			320/ —	166/ —	
	*Wild-type*	12 h	TB7/ TB7+glucose	451/ 2338	216/ 705	Schilling et al., 2015
*Neisseria gonorrhoeae* 1291	*Wild-type*	Overnight	IsoVitaleX-supplemented GC broth	1612	542	Post et al., 2017
	ackA			2401	604	
*Borrelia burgdorferi* B31	Wild-type	SP	BSK-II media	104	64	Bontemps-Gallo et al., 2018
	ΔackA			No acetylation		
	Δpta			242	164	
	ackA complemented strain			206	115	
	Δpta complemented strain			103	78	
*Bacillus subtilis* 3610	Wild-type	SP	LB with 1% glycerol (v/v) and 100 μM MnSO_4_	No significant increase or decrease in the overall acetylation level was observed.		Reverdy et al., 2018
	acuA					
	Pta					

acKa, AcP accumulation.

pta, No conversion of acetyl-CoA into AcP.

ΔackA (Ac-P^-^, acetyl-CoA^-^).

Δpta (Ac-P^+^, acetyl-CoA^-^).

acuA, mutant in lysine acetyltransferase.

Exponential growth phase (EP) and stationary phase (SP).

Since AcCoA and AcP are derived from multiple metabolic pathways in microorganisms, it is difficult to establish whether both or only one of these molecules is the acetyl group donor. The bacterium *Borrelia burgdorferi* is advantageous in this regard because acetate metabolism is limited to the mevalonate pathway that synthesizes lipid I for cell wall biogenesis. In this pathway, acetate is converted to AcP by acetate kinase (AckA), which is metabolized to AcCoA by phosphotransacetylase (Pta) (Richards et al., 2015). Analysis by immunoblotting demonstrated that AckA and Pta were not produced in Δ*ack*A and Δ*pta* mutants, respectively. In the *ackA* mutant complemented with a functional *ackA* gene, AckA was expressed at a level greater than that of the wild-type. Surprisingly, in the *pta* mutant complemented with *pta*, Pta was not overexpressed (Richards et al., 2015).

The acetylome analysis of these mutants and their respective complements showed that no acetylation occurred in the Δ*ackA* mutant, which synthesizes neither AcP nor AcCoA. Increased acetylation was observed in the *ackA* complemented strain, while the Δ*pta* complemented strain had acetylation levels similar to the wild type. Lysine hyper-acetylation was found in the Δ*pta* mutant due to AcP accumulation. Together, these results demonstrate that AcP is the primary source of acetylation (Bontemps-Gallo et al., 2018) (**Table 2**).

To establish the metabolic processes that acetylation by AcP regulates, the modified proteins can be analyzed with different software (PANTHER, DAVID, ERGO, and KEEG). Functional analysis of the *E. coli* proteome revealed that elongation factors, most of the ribosomal subunits, and aminoacyl-tRNA ligases are acetylated in an AcP-dependent manner (Kuhn et al., 2014; Post et al., 2017; Christensen et al., 2019b). In *B. burgdorferi*, the acetylated proteins were involved in genetic information, metabolism and transport, protein folding and degradation, detoxification, motility, and chemotaxis (Bontemps-Gallo et al., 2018). Similarly, some metabolic pathways for carbohydrates (glycolysis/gluconeogenesis, pentose phosphate pathway, pyruvate metabolism, and the TCA cycle), fatty acid, and pantothenate metabolism are sensitive to AcP-dependent acetylation (Kuhn et al., 2014; Schilling et al., 2015).

### 3.2 Characteristics of lysine residues sensitive to chemical acetylation

**Figure 2:**
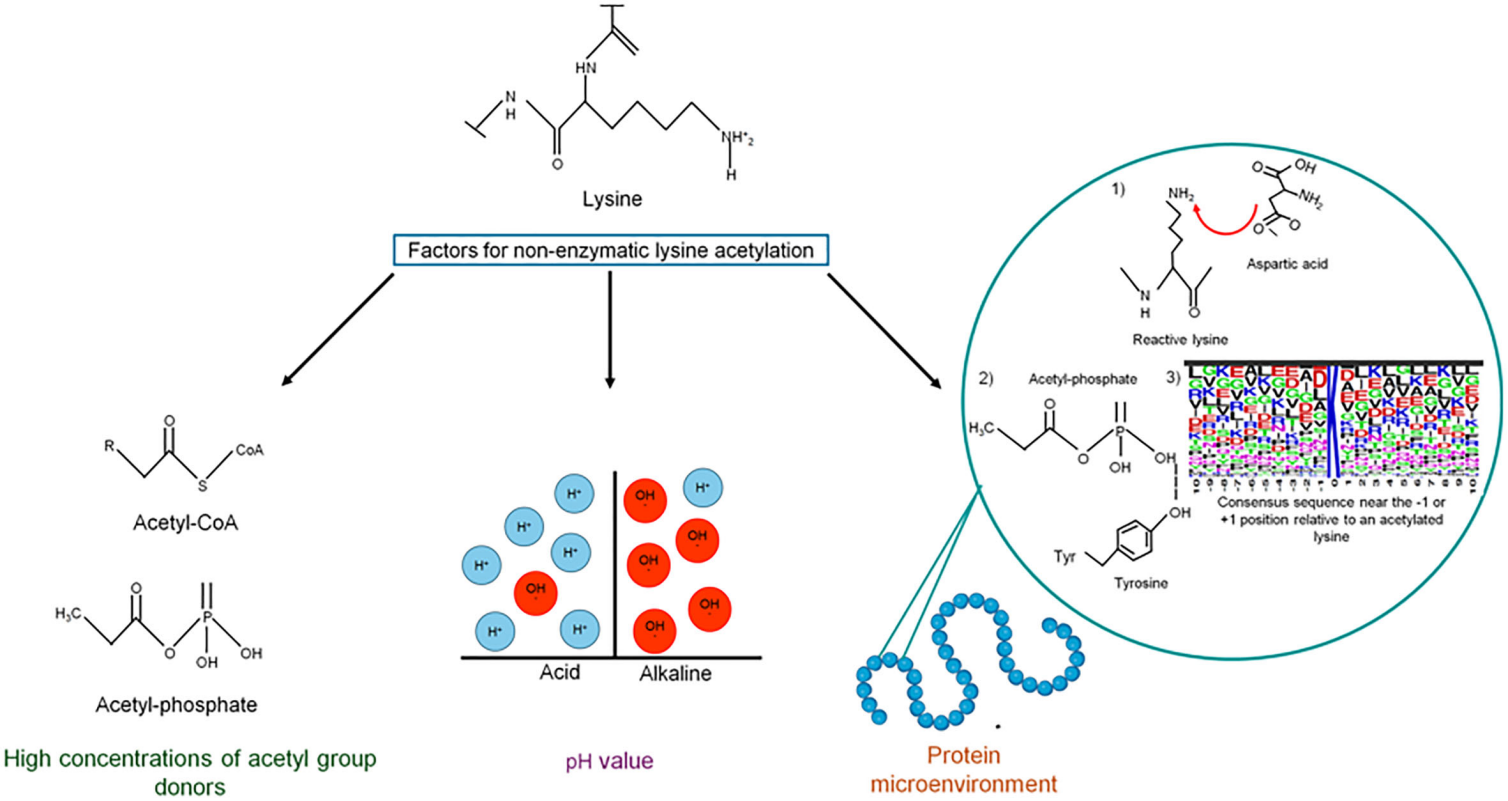
Factors contributing to non-enzymatic acetylation. Acetyl-CoA and acetyl-phosphate are very reactive molecules that, when increasing intracellular concentrations, can non-enzymatically acetylate lysine residues on many proteins. The pH value also plays an essential role in chemical acetylation since, at basic pH, deprotonation of the lysine side chain is favored, increasing its nucleophilicity. In addition to these factors, the efficiency of chemical acetylation also depends on the microenvironment of the protein.

As mentioned, non-enzymatic acetylation is favored by the physicochemical conditions in mitochondria, where the basic pH causes lysine to be deprotonated, which increases its nucleophilicity (**Figure 2**). However, the cytoplasmatic pH in bacteria is maintained at near neutrality: the cytoplasmic pH in neutrophils is ~7.5–7.7, alkaliphiles maintain a basic internal pH of 7.5–8.3, and the pH of acidophilic bacteria is close to 6.5 (Slonczewski et al., 2009; Krulwich et al., 2011). Under these conditions, the epsilon amino groups of most lysine side chains are protonated due to their high pKa and are not very reactive.

For chemical acetylation, lysine reactivity depends on the protein’s microenvironment. First, the presence of negatively charged amino acids (i.e., Asp or Glu) or water molecules deprotonates the reactive lysine. Second, residues with positive charges (Lys or Arg), hydroxyls (Ser, Thr, or Tyr), or amide groups (Gln or Asn) coordinate AcP binding through ionic and hydrogen bonds, which allows the nucleophilic attack of the active lysine on the phosphoryl group of AcP (Hebert et al., 2013; Wagner and Payne, 2013; Kuhn et al., 2014). A tendency to have negatively charged glutamate (E) and/or aspartate (D) residues near the -1 or +1 position relative to an acetylated lysine has been observed, which reduces the pKa of lysine and enhances its reactivity (Kuhn et al., 2014; Post et al., 2017; Christensen et al., 2019b). If the local pH around a lysine is basic, the epsilon amino group can act as a nucleophile toward AcP (Christensen et al., 2019b).

## 4 Deacetylation

Enzymatic or non-enzymatic lysine acetylation can be reversed by lysine deacetylase (KDAC), also known as histone deacetylases (HDAC), since histones were the first identified substrate of these enzymes (Inoue and Fujimoto, 1969; Starai et al., 2002; Gardner et al., 2006). There are two types of KDAs: Zn^2+^-dependent deacetylases and NAD^+^-dependent sirtuins (Gregoretti et al., 2004; Lombardi et al., 2011; Hentchel and Escalante-Semerena, 2015). Bacteria encode putative homologs of both families, but not all function as true deacetylases (Hildmann et al., 2007; Hentchel and Escalante-Semerena, 2015; Christensen et al., 2019b). The first metal-dependent and NAD-dependent deacetylases were described in *Bacillus subtilis* and *Salmonella enterica* serovar Typhimurium, respectively. In both organisms, the acetylation/deacetylation system regulated the activity of the central metabolic enzyme, acetyl-coenzyme A synthetase (Acs) (Tsang and Escalante-Semerena, 1998; Starai et al., 2002, Starai et al., 2004; Gardner et al., 2006).

### 4.1 Sirtuins

**Figure 3:**
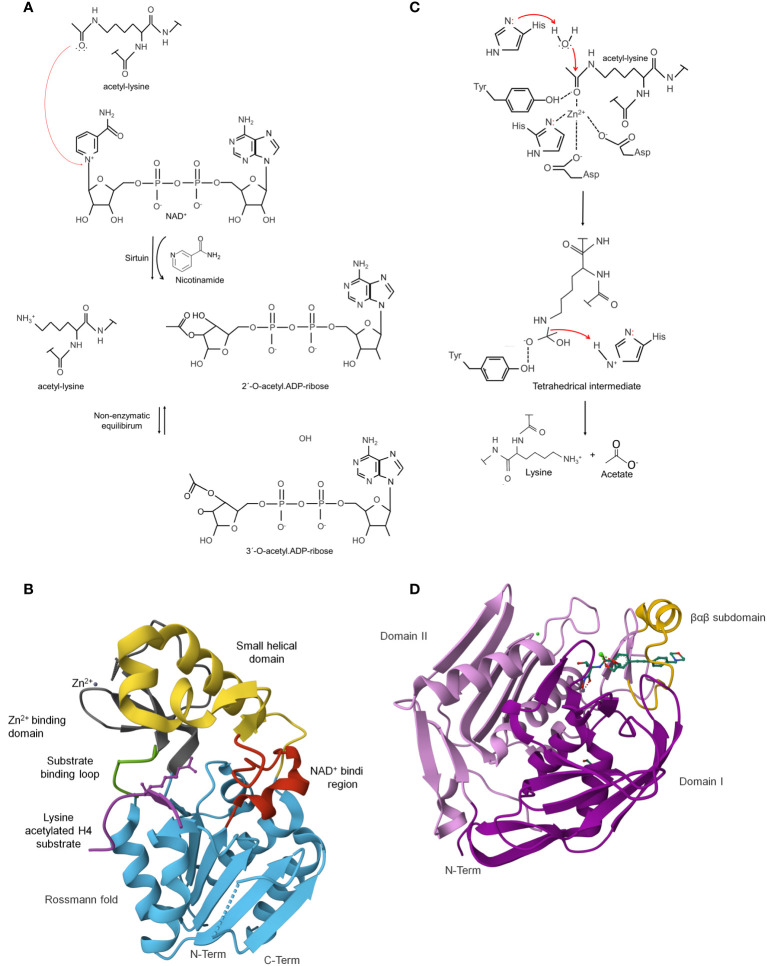
Lysine deacetylases (KDAC) can reverse enzymatic or non-enzymatic lysine acetylation. Catalytic mechanisms used by sirtuins: **(A)** Sirtuins are NAD^+^-dependent lysine deacetylases as a co-substrate for catalysis. In the reaction, the carbonyl-oxygen of the acetyl group of lysine performs a nucleophilic attack on the electrophilic C-1’ of the NAD^+^ ribose, resulting in the fast release of nicotinamide and the formation of a C-1´-*O*-alkylamidate intermediate. The intermediate is hydrolyzed, forming the deacetylated lysine and 2´-*O*-acetyl-ADP-ribose in a non-enzymatic equilibrium with 3´-*O*-acetyl-ADP ribose. Sirtuins are NAD+-dependent lysine-deacetylases **(B)**. Crystal structure of*E*. *coli* sirtuin deacetylase CobB in a complex with a lysine-acetylated substrate (PDB: 1S5P). The characteristic secondary structures of these enzymes are shown in different colors (Zhao et al., 2004) [the figure was generated with PyMOL v.2.3.4 (DeLano, 2002)]. Catalytic mechanisms used by classical deacetylases **(C)**. HDACs use a catalytic water molecule coordinated and polarized by the catalytic Zn^2+^ ion. The ion, together with a histidine residue, interacts with the carbonyl oxygen of the acetyl group. The histidine is polarized and oriented by an aspartic residue (Asp). It acts as a general base to deprotonate the water molecule, thereby increasing the nucleophilicity for attacking the carbonyl carbon of the acetyl group. A second histidine, again polarized and oriented by another Asp, acts as electrostatic catalyst. A tetrahedral oxyanion intermediate is formed, which is stabilized by a histidine and the Zn^2+^ ion, to finally release acetate and the deacetylated lysine [figure redrawn and modified from the decomposition of the oxyanionic tetrahedral intermediate [figure redrawn and modified from Ali et al., 2018; Blasl et al., 2021; Lammers, 2021]. Zinc-dependent classical deacetylase (HDAC/KDAC). are metalloenzymes. **(D)** Crystal structure of *Pseudomonas aeruginosa* zinc-dependent deacetylase LpxC in a complex with the potent BB-78485 inhibitor (PDB: 2ves). LpxC domain consists of two homologous domains, I (colored in magenta) and II (colored in purple) (Mochalkin et al., 2008) [the figure was generated with PyMOL v.2.3.4 (DeLano, 2002).

Sirtuins, also known as Sir2 proteins (silent information regulator 2), have a conserved catalytic core domain characterized by its requirement for nicotine adenine dinucleotide (NAD) to catalyze protein deacetylation by transferring the acetyl group from the lysine to NAD^+^, resulting in deacetylated lysine, nicotinamide (NAM) and 2′-O-acetyl-ADP- ribose (**Figure 3A**) (Rine and Herskowitz, 1987). These proteins are evolutionarily conserved within the domains of bacteria, archaea, and eukaryotes (Avalos et al., 2004; Hawse et al., 2008).

Genomes of bacteria and most archaea encode one or two sirtuins, whereas eukaryotes typically contain multiple sirtuins. Lammers (2021) suggests that the low number of sirtuins in bacteria allows them to control specific physiological processes where they can present a very narrow substrate range or may have a high degree of promiscuity in substrate recognition. An enzyme with high substrate promiscuity may also have specificity for certain substrates because they are processed more efficiently due to the region within the cell where the reaction occurs or by transcriptional regulation of their expression levels (Lammers, 2021).

#### 4.1.1 Overall structure of sirtuin

The alignment of sirtuin primary sequences shows a highly conserved catalytic core, while the N- and C-terminal regions are variable in length and sequence (Yuan and Marmorstein, 2012).

The catalytic core adopts an elongated shape containing a conserved large Rossmann-fold domain and a smaller and more structurally diverse domain for acyl peptide and NAD+ binding. These domains are connected by a series of loops that contribute to forming a cleft between the large and small domains (North and Verdin, 2004; Zhao et al., 2004; Sanders et al., 2010).

The large domain comprises an inverted prototypical open α/β Rossmann fold structure, widely distributed in proteins that bind to NAD in its oxidized (NAD^+^) or reduced (NADH) form. This domain comprises six parallel β strands forming a central β sheet packed between several α helices. The exact number of α helices depends on the protein. For example, *E. coli* sirtuin CobB contains eight α helices. Also, a conserved Gly-X-Gly sequence important for phosphate binding, a pocket to accommodate an NAD+ molecule, and charged residues responsible for ribose group binding are found (**Figure 3B**) (Sanders et al., 2010).

The structural Zn^2+^-binding domain is composed of three antiparallel β-strands and a variable α helical region. A zinc ion is generally bound to four conserved lysine residues in the β-sheet module in a tetrahedral conformation, except for CobB, where it is linked to two cysteine residues and contains three of the four expected zinc-coordinating cysteine residues according to sequence alignment (Sanders et al., 2010; Yuan and Marmorstein, 2012). The zinc ion does not participate directly in the deacetylation but has an essential structural role in the integrity of the catalytic core domain (**Figure 3B**) (Blander and Guarente, 2004; Yuan and Marmorstein, 2012).

A binding site located between the sirtuin large and small domains, linked to each other by two flexible loops (L1 and L2), forms a cleft that acts as the enzyme’s active site. NAD+ and acetyl-lysine substrate bind at this cleft (Yuan and Marmorstein, 2012). The binding region is divided into three spatially distinct sites: site A, for an adenine-ribose moiety of NAD binding; Site B, for the nicotinamide-ribose moiety binding; and Site C site is located deep in the NAD-binding pocket for nicotinamide moiety binding (**Figure 3B**) (Sanders et al., 2010).

### 4.2 Zinc-dependent deacetylases

Metal-dependent HDACs are hydrolases that cleave the acetyl group from lysine to yield free lysine and acetate (**Figure 3C**). They belong to many proteins, including acetylpolyamine amidohydrolases, acetoin utilization proteins, and histone deacetylases (Hernick and Fierke, 2006).

These enzymes are classified in eukaryotic organisms based on their homology, domain organization, and cellular localization with respect to yeast deacetylases. Class I HDACs are closely related to the transcriptional regulator RPD3 of *Saccharomyces cerevisiae*, with a length of 350–500 amino acids. They are principally localized in the nucleus and have a variable C-terminus with nuclear import and export signals. Class II HDACs are about 1,000 amino acids long, with a catalytic domain containing several conserved sequence motifs. These enzymes are primarily localized in the cytoplasm, have unique binding sites at their N-termini to control translocation of the protein in and out of the nucleus in response to specific cellular signals, and thus are at least in part cytoplasmic and, in some cases, act on non-histone protein substrates (Ruijter et al., 2003; Hildmann et al., 2006; Wagner et al., 2013). Class IV contains only a single enzyme localized to the nucleus.

In bacteria, some proteins have been identified as members of the family of zinc-dependent deacetylases. For example, the LpxC in gram-negative bacteria (*E. coli, Aquifex aeolicus*, and *Pseudomonas aeruginosa*) (Jackman et al., 2000; Whittington et al., 2003; Hernick and Fierke, 2006);, PA3774 of *Pseudomonas aeruginosa* (Majorek et al., 2013), AcuC of *Bacillus subtilis* and *Aeromonas hydrophila* (Gardner et al., 2006; Jiang et al., 2017), FB188 HDAH (histone deacetylase-like amidohydrolase) from *Bordetella*/*Alcaligenes* strain FB188 (Hildmann et al., 2004; Nielsen et al., 2005) and LdaA of *Rhodopseudomonas palustris* (Crosby et al., 2010).

#### 4.2.1 Overall structure of zinc-dependent deacetylases

The HDAC structure is characterized by an α/β fold topology (Yang and Seto, 2008; Hentchel and Escalante-Semerena, 2015). In some family members, crystallography shows that one or two domains form the tertiary structure. For example, in the LpxC proteins, the two domains have an identical topology of secondary structural elements that includes a five-stranded parallel β-sheet and two principal α-helices connected by a 16-residue linker (Whittington et al., 2003). In contrast, the HDAC8 comprises a single domain consisting of an eight-stranded parallel β-sheet sandwiched between 13 α-helices (**Figure 3D**) (Somoza et al., 2004). Interestingly, the crystal structure of PA3774 from the human pathogen *P. aeruginosa* shares a high degree of homology with class IIb HDACs and consists of two dimers that are close to each other, forming a tetramer, which may be essential for substrate recognition and selectivity (Kraümer et al., 2016). Despite these differences, the structural comparison shows that the structural difference is mainly restricted to the loop regions.

The catalytic center contains a zinc ion commonly pentacoordinated by two aspartic acids, a histidine, and a water molecule. In addition to the zinc ligands, two histidine, two aspartic acids, and one tyrosine form hydrogen bonds with bound ligands. Mutation of any of these residues completely abolishes activity (Kraümer et al., 2016). Furthermore, the surface of this site reveals the formation of a narrow pocket that probably serves to accommodate the acetylated lysine during the catalytic reaction (Vannini et al., 2004). The reaction requires that a conserved histidine residue acting as a general base to activate a metal-bound water that attacks the carbonyl of the acetyl group (**Figure 3D**) (Finnin et al., 1999; Finnin, 2005; Hernick and Fierke, 2006).

## 5 Cellular processes regulated by acetylation

### 5.1 Bacterial metabolism

**Figure 4:**
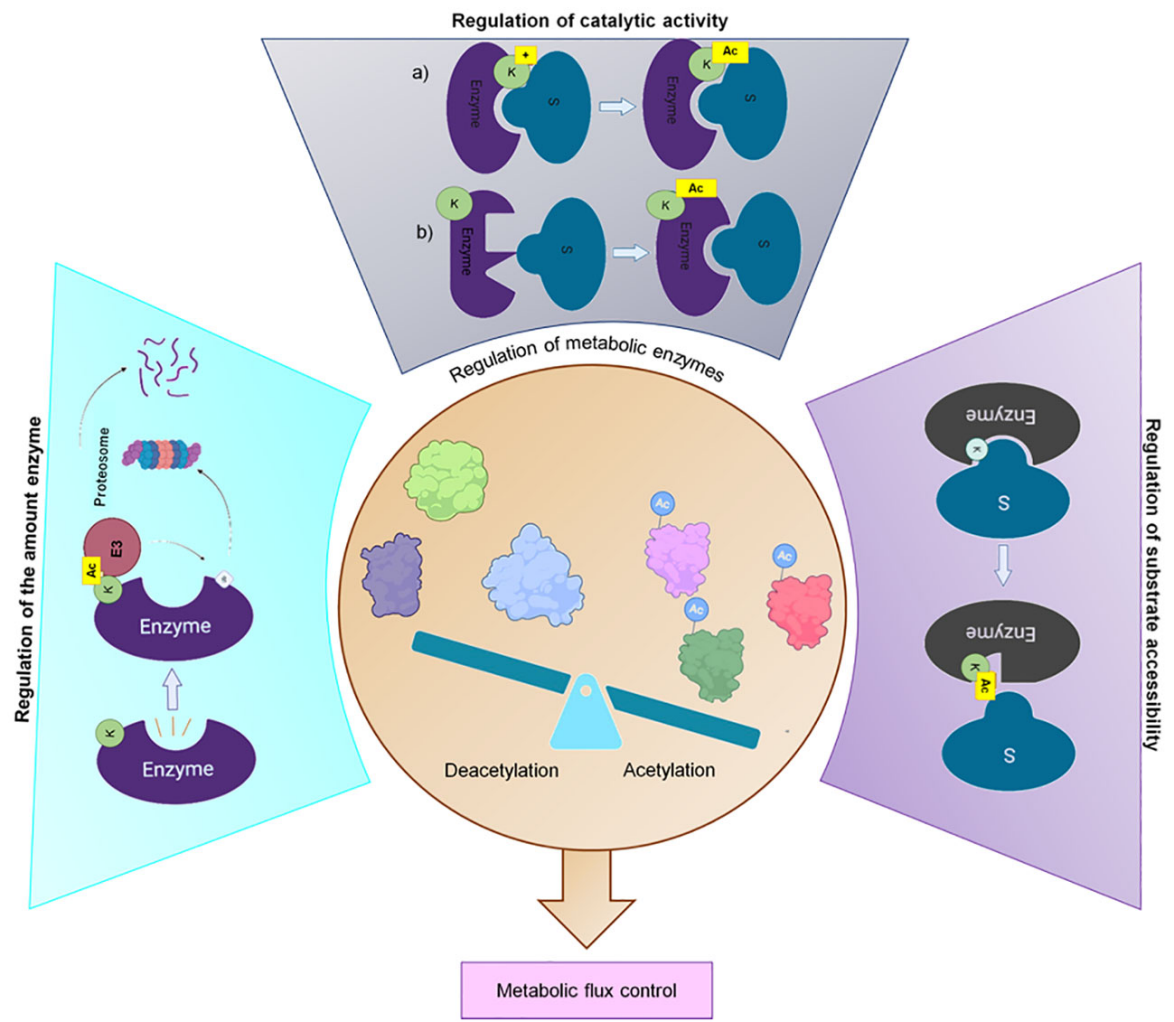
Regulation of the metabolic enzyme activity. Acetylation regulates 1) The number of metabolic enzymes by promoting their degradation through the ubiquitin–proteasomal system, 2) The catalytic activity through a) neutralizing the positive charge of lysine residues in the active site or b) causing allosteric changes and 3) The substrate accessibility to metabolic enzymes by modifying the conserved lysine residues to hinder the entry of substrate (Xiong and Guan, 2012; Liu et al., 2021). Created with BioRender.com.

To survive and compete in their natural habitats, bacteria must be able to respond to environmental disturbances or nutrient fluctuations by adapting their metabolism. A quick response mechanism is regulated by the activity of metabolic enzymes, which can be controlled by the amount of enzyme, the catalytic activity, and the substrate accessibility (**Figure 4**) (Xiong and Guan, 2012). Reversible post-translational modification, such as lysine acetylation, may be involved in at least one of these aspects.

Acetylation regulates the amount of an enzyme by promoting the assembly of functional multimeric structures that bind to the proteasome for the degradation of proteins or by targeting the substrate for ubiquitylation and proteasome-dependent degradation (Xiong and Guan, 2012; Liu et al., 2021). The enzymatic activity regulation is related to changes in the physical-chemical properties of the catalytic pocket, which could inhibit substrate accessibility (Kim et al., 2006; Nakayasu et al., 2017). In this regard, Nakayasu and co-workers demonstrated that some glycolytic and TCA cycle enzymes are acetylated on highly conserved lysine residues located within the catalytic pocket of the enzyme. This modification dramatically alters the charge and shape of the lysine residue by neutralizing its positive charge and increasing its size, which inhibits enzymatic activity (Nakayasu et al., 2017).

### 5.2 Regulation of enzymatic activity

In bacteria, the role of N-epsilon lysine in regulating enzymatic activity was first reported in the Acetyl-CoA synthetase (Acs) of *S. enterica* (Starai et al., 2002). *In vivo* and *in vitro* assays showed that the acetyltransferase Pat interacts with leucine 641 of Acs, leading to acetylation of lysine-609, decreasing Acs’s activity—incubation of the acetylated enzyme with NAD^+^-dependent deacetylase CobB results in its activation. A Leu-641 Acs mutant showed that position 641 has a structural contribution that allows the interaction of Acs with the protein acetyltransferase (Pat) (Starai et al., 2002; Starai and Escalante-Semerena, 2004b; Starai et al., 2004). In other bacteria (*E. coli, B. subtilis, Saccharopolyspora erythraea, Rhodopseudomonas palustris, M. smegmatis and Staphylococcus aureus*) similar acetylation-dependent regulatory modes of Acs have been described (Gardner et al., 2006; Xu et al., 2011; Crosby et al., 2012; Kim et al., 2013b; You et al., 2014; Castaño-Cerezo et al., 2015; Burckhardt et al., 2019). Acetylation also negatively regulates the isocitrate dehydrogenase (ICDH) activity from *Mycobacterium tuberculosis.* The enzyme is acetylated in two lysine residues by Rv2170, reducing the enzymatic activity to around 30% (Lee et al., 2017).

As demonstrated by Venkat et al. (2017a), protein acetylation can also increase enzyme activity. The enzymatic activity of different acetylated variants of malate dehydrogenase (MDH) obtained by an expansion of the genetic code strategy showed that only the acetylation at positions K99 and K140 and the MDH acetylated at both positions increased the enzyme activity. In other variants, no effect was observed. The authors also demonstrated that MDH acetylation can occur either enzymatically or non-enzymatically and that the level of MDH acetylation increases in a glucose-dependent manner (Venkat et al., 2017a). The glyceraldehyde-3-phosphate dehydrogenase (GAPDH), an essential enzyme in glycolysis and gluconeogenesis, catalyzes the oxidation of D-glyceraldehyde-3-phosphate to 1,3-bisphosphoglycerate with NAD+ reduction to NADH. In *E. coli*, acetylation positively regulates the enzyme; incubation of purified GAPDH with the acetyltransferase PatZ led to a two-fold increase in its enzymatic activity (Plekhanova et al., 2023).

In other proteins, the effect on activity depends on the lysine residue that is acetylated. For example, the enzyme assay and kinetic analysis of different acetylated variants of *E. coli* citrate synthase (CS) showed that lysine acetylation could decrease the overall CS enzyme activity, mainly due to the acetylation of K295, which impaired the binding of acetyl-coenzyme A. However, acetylation at K283 increased the enzymatic activity since the binding of acetyl-coenzyme A is promoted (Venkat et al., 2019). A similar result was observed in the isocitrate dehydrogenase (ICDH) (Venkat et al., 2018a). Acetylation at K55 or K350 of ICDH significantly increased the activity, while acetylation at K235 caused a loss of about 40% of the activity, and acetylation at K100 or K230 almost eliminated activity (Venkat et al., 2018a). For lactate dehydrogenase (LdhA), acetylation levels for some lysine residues correlate with enzymatic activities. Residues K154 and K248 present low acetylation levels and a decrease of approximately 30% in enzymatic activity. However, residue K9 also showed the same decrease in the acetylation levels, but its enzymatic activity increased 2.5-fold. According to the 3D-modelled structure of LdhA, K154 may be involved in binding the substrate and cofactor, while K248 and K9 are not the catalytic key sites, but both help stabilize the LdhA conformation (Liu et al., 2022).

Reversible lysine acetylation (RLA) has been demonstrated for some of these enzymes. In *Salmonella enterica*, Acs’s activity can be restored by the NAD+-dependent CobB sirtuin deacetylase, which removes the acetyl group from acetylated Acs (Starai et al., 2002; Starai and Escalante-Semerena, 2004b; Starai et al., 2004, Starai et al., 2005). For *B. subtilis*, Acs’s activity is restored by two deacetylases, AcuC and SrtN; the first does not require NAD^+^, while the second is a class III NAD+-dependent sirtuin deacetylase (Gardner et al., 2006; Gardner and Escalante-Semerena, 2009). This suggests that the regulatory mechanisms of Acs activity in these two bacteria differ. For both bacteria, inactivation of the enzyme by acetylation produced a growth defect on acetate, which can be restored by deacetylation of the enzyme. However, *B. subtilis* could respond to different cell energy charge indicators. On the one hand, the activation by AcuC may be triggered in response to a low AcCoA: CoA ratio. On the other hand, if the NAD+:NAD ratio H becomes high, the SrtN-dependent is activated. In *Salmonella enterica*, the mechanism seems to be more straightforward. Acs synthesizes AcCoA in two steps, the first in which the acetate is adenylated and a subsequent thioester formation step. In response to high levels of AMP due to the uncontrolled activity of the enzyme, acetylation of K609 blocks the adenylation reaction and stops the synthesis of acyl-AMP intermediates without altering the thioester-forming activity (Starai et al., 2002; Starai and Escalante-Semerena, 2004a; Starai et al., 2004, Starai et al., 2005; VanDrisse and Escalante-Semerena, 2019). The tight regulation of the enzyme by the acetylation/deacetylation mechanism occurs because high levels of AMP in the cell indirectly lead to lower levels of ATP synthesis (VanDrisse and Escalante-Semerena, 2019). In addition, the cell could use this mechanism to maintain the acetate pool and prevent unnecessary ATP hydrolysis (Starai et al., 2003; Chan et al., 2011). We highly recommend reading the article of Hentchel and Escalante-Semerena, which discusses the RLA in gram-negative and gram-positive bacteria in depth.

It has also been shown that CobB sirtuin has site-specificities. Most of the CobB-sensitive acetylated lysine sites are located on the surface of the protein, while CobB-resistant sites are in regions of the protein where the enzyme does not have easy access. For example, some acetylated lysines are located at the dimer interface or are deeply buried near the active site, limiting the access of CobB (Fatema and Fan, 2023).

These studies prove that acetylation modulates the activity of central metabolic enzymes and eventually alters protein function to control competing pathways in response to physiological needs.

### 5.3 Carbon source-regulated protein acetylation

Comparative studies with different carbon sources have explored the effect of protein acetylation during growth under glycolytic and oxidative conditions. As Wang and collaborators demonstrated, *S. enterica* presents changes in cell growth and significant differences in the percentage of acetylated proteins in response to glucose or citrate. A total of 15 enzymes were identified with altered acetylation status in response to a carbon source, and all of them showed higher acetylation levels in cells grown in glucose (Wang et al., 2010). The main targets of lysine acetylation were enzymes involved in central metabolism, and a correlation was observed in the cell growth and the acetylation levels of these proteins. In a medium with glucose, cells grow faster, and an increase in the acetylation of central metabolic enzymes was observed, but when the acetylation decreases, opposite growth properties were observed (Wang et al., 2010). In *B. subtilis*, different acetylation levels have also been reported in different carbon sources. In decreasing order, protein acetylation occurs in a medium containing only carbon source pyruvate, glucose, glycerol, or citrate (Kosono et al., 2015). The main differences in the acyl modification patterns were observed in glucose and citrate. Under these two conditions, changes in the acetylation sites were determined. Acetylation was upregulated at 13 sites in glucose relative to citrate, and, as in *S. enterica*, acetylation positively modulated the growth of this bacterium in a glucose medium (Kosono et al., 2015). Western immunoblot analysis found that glucose and lactate produce global acetylation in *E. coli* K-12, which increased after the cells had entered the stationary phase. Induction of acetylation at this growth point requires continued exposure to carbon sources, specifically glucose (Schilling et al., 2015). The results suggest that protein acetylation may have a physiological role in mediating adaptation to different carbon sources.

Another factor that could determine lysine acetylation levels is the sugar concentration. In *E. coli*, the increase in acetylation levels occurs during growth at high concentrations of glucose or xylose (4% for both carbon sources), compared to low concentrations of 0.4%. No changes in the profiles associated with the type of carbon source were observed, suggesting that the sensitivity of lysines to acetylation depends on the amount of sugar available in the medium. Furthermore, it was found that significantly more acetate is produced under these conditions, and *E. coli* cannot consume it. The results support the hypothesis that most acetylation results from acetate overflow metabolism and is independent of the specific catabolic route (Schilling et al., 2015, Schilling et al., 2019).

### 5.4 Regulation of acetate metabolism by protein acetylation

The concept of metabolic overflow refers to a phenomenon that occurs during growth at a high concentration of carbon source or during the transition from a limited substrate condition to a rich one. Under these conditions, cells exhibit a metabolic shift characterized by the secretion of many primary metabolites, such as acetate, ethanol, or lactate, even in the presence of oxygen. This occurs when the rate of the substrate (e.g., glucose) uptake exceeds the capacity of the cell´s respiratory machinery to fully oxidize it via the TCA cycle and oxidative phosphorylation (Pinu et al., 2018).

Metabolic overflow has been studied in the model microorganisms *E. coli* and *S. cerevisiae*. In *E. coli*, acetate overflow is observed when cells are grown aerobically in high glucose concentrations (Paczia et al., 2012; Pinu et al., 2018). Under these conditions, there is an imbalance between the fluxes of glucose uptake, energy production, and carbon conversion into biomass and products, causing the excretion of significant amounts of acetate (Bernal et al., 2016). The excreted acetate can be used after the depletion of the primary carbon source by the acetyl-CoA synthetase (Acs), which converts acetate to acetyl-coenzyme A (Ac-CoA), which is mainly metabolized by the glyoxylate shunt and the TCA cycle (Starai and Escalante-Semerena, 2004a; Castaño-Cerezo et al., 2015). Acs is tightly controlled at the transcriptional level, and its activity, as mentioned, is posttranslationally regulated by protein acetyltransferase.

The role of the regulation of Acs by the acetylation/deacetylation mechanisms in acetate metabolism was first described in *Salmonella enterica*, where it was observed that acetyltransferase Pat acetylates Acs inhibiting its activity and the sirtuin deacetylase CobB reactivate the Acs enzyme, restoring growth on acetate (10 mM) as the sole carbon source (Starai et al., 2002, Starai et al., 2003; Starai and Escalante-Semerena, 2004b; Starai et al., 2004, Starai et al., 2005; VanDrisse and Escalante-Semerena, 2019).

In *E. coli* and *Mycobacterium smegmatis*, acetate metabolism is also regulated by the acetylation/deacetylation of Acs (Castaño-Cerezo et al., 2011; Hayden et al., 2013; Castaño-Cerezo et al., 2014; Peebo et al., 2014). Studies in *E. coli* have demonstrated that deleting the NAD+-dependent deacetylase (ΔcobB) strongly affects its physiology when grown in a minimal medium with acetate or glucose as the only carbon source. First, the bacterium is unable to grow at low acetate concentrations (10 mM). Secondly, the mutant strain exhibits a high glucose consumption rate in glucose cultures, leading to more significant acetate accumulation in the medium and slower acetate absorption (Castaño-Cerezo et al., 2011). Under glucose-limited conditions, an increase in acetylation of Acs is observed, with activity decreasing to almost half that of the wild‐type (Castaño-Cerezo et al., 2014). These results support the idea that the inactivation of Acs by acetylation affects the high-affinity Acs pathway for acetate assimilation, which primarily serves to scavenge acetate at low concentrations. Additionally, it has been hypothesized that the regulation of the isocitrate node involves two post‐translational modifications, phosphorylation and acetylation, each operating at distinct levels. AceK is proposed to oversee a broad regulatory mechanism that impedes the flux through the TCA cycle. Meanwhile, acetylation is suggested to contribute to the fine-tuning regulation of the glyoxylate shunt, partially inhibiting its activity. This partially inhibited glyoxylate shunt activity could explain the reduced growth rate observed in the Δ*cobB* mutant under acetate and glucose-limited conditions (Castaño-Cerezo et al., 2011, Castaño-Cerezo et al., 2014).

Furthermore, acetylation of Acs may be involved in controlling the co-utilization of fermentable substrates. A mutation in Leu-641 makes Acs insensitive to acetylation even at high glucose concentrations (Starai et al., 2005). The overexpression of this protein in *E. coli* W allows the efficient co-utilization of glucose and acetate. In a batch process containing glucose and high acetate concentrations, a 2.7-fold increase in acetate uptake was observed in comparison to a control strain (Novak et al., 2018).

### 5.5 Regulation of metabolic flux

Most microorganisms have developed different strategies to sequentially metabolize a mixture of simple carbohydrates, favoring the utilization of glucose as a carbon and energy source to sustain a higher growth rate. The metabolic plasticity allows them to obtain specific carbon sources efficiently and survive in competitive environments (Vinuselvi et al., 2012). Most bacteria regulate their metabolism via carbon catabolite repression (CCR), which involves a complex interplay between metabolism, signaling by proteins and metabolites, and the regulation of gene expression (Kremling et al., 2015). Alternative splicing, mRNA stability, translation, and protein degradation control of enzymes are other mechanisms that can be used to modulate carbon flux. These coarse regulations usually respond to long-term environmental changes (Wegner et al., 2015).

Post-translational modifications affect flux distribution between important metabolic branches, such as glycolysis and gluconeogenesis, TCA cycle and glyoxylate shunt, and glycolysis and TCA cycle. Wang et al. (2010) demonstrated that carbon source-associated acetylation modulates metabolic flux profiles in *S. enterica*. In the presence of glucose, acetylation increases the glycolysis/gluconeogenesis flux ratio 2.07-fold, while acetylation reduces the glyoxylate bypass/TCA flux ratio under a citrate-based carbon source.

The isocitrate node is a vital regulation point of carbon flux between the TCA cycle and the glyoxylate shunt. Isocitrate, the substrate of isocitrate dehydrogenase (ICDH) and isocitrate lyase (AceA), is converted to α-ketoglutarate by ICDH or is cleaved to succinate and glyoxylate by AceA, directing the carbon source flow to TCA cycle or to glyoxylate shunt, respectively. In the 70s, Holms and Bennett, (1971) were the first to report the inactivation of ICDH at the end of the growth of *E. coli* on glucose, and later, this same behavior was reported when *E. coli* was grown on acetate as the only carbon source (Bennett and Holms, 1975). The authors also demonstrated that when pyruvate, malate, or succinate were added to cultures growing on acetate, the ICDH activity increased (Bennett and Holms, 1975). The inhibition of ICDH enzymatic activity was attributed to phosphorylation, which decreases isocitrate flux to the TCA cycle and forces it through the glyoxylate bypass (Garnak and Reeves, 1979). The phosphorylated serine residue at position 113 completely inactivates the enzyme, while dephosphorylation restores the impaired activity (Dean et al., 1989). Although historically, it was considered that phosphorylation was the only way to regulate the enzyme, proteomic studies have demonstrated that metabolic enzymes, including ICDHs, are favorable targets for lysine acetylation (Xu et al., 2017). In *E. coli*, proteomic studies have identified more than 20 acetylated lysine residues in ICDH (Yu et al., 2008; Zhang et al., 2009; Weinert et al., 2013) and, as mentioned in the regulation of enzymatic activity section, ICDH activity in *in vitro* assays can be regulated by lysine acetylation. However, it would have to be demonstrated that this phenomenon also occurs *in vivo*. In *Salmonella enterica* serovar Typhimurium ICDH is also regulated by phosphorylation (Wang and Koshland, 1982).

In *M. tuberculosis*, this metabolic node is regulated by the acetylation of the ICDH. Acetylation suppresses enzyme activity in the presence of fatty acids, reducing carbon flow into the TCA cycle (Lee et al., 2017). The activation of glyoxylate bypass allows the conversion of AcCoA to the metabolic intermediate succinate to support the growth in the presence of non-carbohydrate substrates such as fatty acids or acetate (Cronan and Laporte, 2005; Lee et al., 2017).

Recently, it was demonstrated that glyceraldehyde 3-phosphate dehydrogenase (GapA) and 2,3-bisphosphoglycerate-dependent phosphoglycerate mutase (GpmA) were sensitive to non-enzymatic acetylation *in vitro* at physiological AcP concentrations. In both enzymes, acetylation reduced their activity, which could be reflected in reduced glycolytic/gluconeogenic flux in conditions with higher concentrations of AcP (Schastnaya et al., 2023).

The flux from glucose to glutamate is increased when the cell excretes glutamate. Factors like the depletion of biotin and the addition of detergents or antibiotics trigger glutamate overproduction and, therefore, a change in the flux of central carbon metabolism to favor glutamate production (Shirai et al., 2007). It has been proposed that in addition to the decrease in 2-oxoglutarate dehydrogenase complex (ODHC) activity, the regulation of phosphoenolpyruvate carboxylase (PEPC) activity by acetylation may be a mechanism involved in the change in metabolic flux during overproduction of glutamate. PEPC catalyzes the irreversible carboxylation of phosphoenolpyruvate to generate oxaloacetate, ensuring that the carbon flow is directed toward glutamate production via the TCA cycle. Acetylation at the K653 site regulates enzyme activity and is, therefore, the mechanism that maintains metabolic flux under glutamate-producing conditions (Mizuno et al., 2016; Nagano-Shoji et al., 2017).

Hence, acetylation may provide a new strategy for regulating protein activity and improving the utilization of different carbon sources.

## 6 Role of acetylation in bacterial pathogenicity

### 6.1 Antibiotic resistance

Although the use of antibiotics to treat infectious diseases has improved quality of life and reduced mortality, their excessive, unnecessary, and incorrect usage has caused the rapid emergence of antibiotic-resistant strains. It is estimated that antibiotic resistance is a growing problem that accounts for approximately 1.27 million global deaths (Tacconelli et al., 2018; WHO, 2023). Furthermore, this number is expected to increase in the coming years, leading cause of 10 million deaths worldwide by 2050 (Zalewska-Piątek, 2023). Understanding the antibiotic resistance mechanisms in bacteria will allow the development of new antimicrobial agents with novel mechanisms of action or new strategies for innovative non-antibiotic therapeutic methods.

Bacteria have developed sophisticated molecular strategies to counteract the effect of antibiotics; for example, antibiotic sequestration by specific proteins can prevent them from reaching their targets. Increased efflux pump activity to extrude the antibiotic or decreased influx to avoid antibiotic entry reduces the effective concentration of antibiotics and, therefore, decreases their efficiency (Luther et al., 2018). Finally, bacteria can use enzymatic strategies to inactivate antibiotics. In this mechanism, the antibiotic can be degraded by the action of β-lactamases, macrolide esterases, and epoxidases or can be covalently modified. These chemical modifications include O-phosphorylation, O-nucleotidylation, O-ribosylation, O-glycosylation, O-acetylation, and N-acetylation (Wright, 2005).

Aminoglycosides are a large family of antibiotics with broad-spectrum used to treat Gram-negative and Gram-positive bacterial infections (Magalhaes and Blanchard, 2005). Aminoglycosides inhibit bacterial protein synthesis by binding to the aminoacyl-tRNA site (A-site) of 16S rRNA, resulting in an erroneous reading and/or hindering the translocation step (Magnet and Blanchard, 2005; Barrett et al., 2008; Vetting et al., 2008). The emergence of a growing number of resistant strains has limited its use. One of the principal causes of aminoglycoside resistance is related to its enzymatic modification by aminoglycoside N-acetyltransferases (AACs), proteins members of the GNAT superfamily.

Genes encoding aminoglycoside N-acetyltransferases have been described in pathogenic bacteria. AACs catalyze the acetyl-CoA-dependent N-acetylation of the amino group of aminoglycosides, inhibiting their interaction with the 16S rRNA, resulting in microbial resistance and complications in the clinical treatment (Teran et al., 1991; Shaw et al., 1992; Costa et al., 1993; Magnet et al., 1999; Vetting et al., 2004; Magnet and Blanchard, 2005). ACCs selectively transfer the acetyl group to one of the four amino groups of the typical aminoglycoside structure. According to this regiospecificity, these enzymes are grouped into four major classes: AAC(1), AAC(3), AAC(2′), and AAC(6′) (Draker and Wright, 2004; Magalhaes and Blanchard, 2005; Magnet and Blanchard, 2005; Wright, 2005). However, not all the AACs are related to aminoglycoside resistance; the decreased binding of the aminoglycoside to the A-site is only associated with the acetylation at the position three′ of the 2-deoxystreptamine ring by AAC(3) or the acetylation at position 6′of the amino hexose by AAC(6′) of the aminoglycoside (Magnet and Blanchard, 2005; Barrett et al., 2008).

Interestingly, some clinical isolates of Gram-negative bacteria contain a variant of an aminoglycoside-modifying enzyme, the aminoglycoside acetyltransferase AAC(6′)-Ib, which has two amino acid substitutions, Trp102Arg and Asp179Tyr, that are not found in other variants (Park et al., 2006; Robicsek et al., 2006; Jung et al., 2009; Kim et al., 2013a). In particular, the Asp179Tyr mutation allows the enzyme to acetylate fluoroquinolones, representing an alternative resistance mechanism for this synthetic drug (Vetting et al., 2008).

Recently, it has been found that lysine acetylation of proteins involved in essential metabolic processes allows the bacteria to respond to antibiotic stress, which can significantly affect the bactericidal effect of antibiotics. An acetylome analysis of *E. coli* strains resistant to three types of antibiotics (ampicillin, kanamycin, or polymyxin B) showed a common regulatory mechanism. Acetylation positively regulates bacterial motility by reducing the acetylation of flagellar proteins. It negatively regulates energy metabolism by increasing the acetylation levels of central metabolic proteins. This reduces the metabolic level of bacteria to regulate antibiotic resistance. Lower levels of acetylation cause downregulation of the ribosome, RNA degradation, signal transduction, and protein export, which could inhibit signal transduction and prevent the antibiotic from binding to the target protein (Fang et al., 2022). Also, the KEGG analysis of lysine acetylation shows enrichment of this post-translational modification in different metabolic processes such as the TCA cycle, pyruvate metabolism, and glycolysis (Fang et al., 2022). Similar results have been found in other pathogenic organisms, demonstrating that central metabolic pathways regulate bacterial antibiotic susceptibility (Peng et al., 2015; Xie et al., 2016; Pang et al., 2020a). However, more studies are necessary to elucidate the correlation between acetylation and antibiotic resistance.

The other way acetylation controls antibiotic response is related to virulence factors. In *Salmonella* Typhimurium, deleting the acetyltransferase *pat* reduced the pathogenicity island 1 (SPI-1) expression, affecting its ability to invade HeLa cells and intramacrophage replication. This mutant has attenuated virulence compared to the mouse model’s wild type. It has been proposed that HilD, a critical transcriptional regulator of SPI-1, could mediate the acetylation-related virulence in *Salmonella*. The acetylation stabilizes this transcriptional regulator and promotes the transcription of HilA, leading to the activation of SPI-1 (Sang et al., 2016). The first *Salmonella* acetylome study identified HilD as an acetylated protein (Wang et al., 2010). In contrast, when HilD is deacetylated, it tends to be degraded and does not activate HilA transcription, lowering SPI-1 expression and leading to attenuated virulence of the bacteria (Sang et al., 2016; Koo et al., 2020).

Another vital virulence factor in *Salmonella* is the PhoP/PhoQ two-component system, which regulates virulence in response to various signals within the mammalian host. PhoP is acetylated and deacetylated *in vitro* and *in vivo* by Pat and CobB, respectively. Specifically, when the conserved lysine residue 201(K201) located in the C-terminal DNA-binding domain of PhoP is acetylated, its ability to bind to DNA is inhibited, altering the transcription of *phoP* and PhoP-regulated genes. This enzymatic acetylation impairs bacterial intracellular replication and inflammation response in macrophages and significantly attenuates the bacteria virulence, suggesting that non-acetylated PhoP is essential for *Salmonella* pathogenesis. With these results, the authors propose that regulating transcriptional factors by acetylation of the DNA binding domain may be a new regulatory mechanism of gene expression involved in bacterial virulence (Ren et al., 2016, Ren et al., 2017).

The global acetylome analysis of different bacteria has allowed the identification of other acetylated virulence factors. In *M. tuberculosis*, the heat-shock protein X (HspX) linked to the latent infection and the isocitrate lyase involved in the persistence, virulence, and antibiotic resistance are acetylated (Liu et al., 2014; Xie et al., 2015). In *Salmonella*, two virulence factors (inositol phosphate and the pathogenicity island one effector protein SipC) are acetylated after the bacteria acquire drug resistance (Li et al., 2018). The virulence factors exotoxin A, chitin-binding protein, serine protease, and hemolysin have been characterized as lysine-acetylated proteins in *P.* aeruginosa (Ouidir et al., 2015). In *Vibrio vulnificus* and *Vibrio alginolyticus*, several virulence factors are found to be acetylated (Pang et al., 2020a, Pang et al., 2020b). For *E. amylovora*, the proteins exopolysaccharide amylovoran biosynthesis- and type III secretion-associated proteins involved in virulence are lysine-acetylated (Zhao et al., 2009; Wu et al., 2013). In *P. aeruginosa*, some virulence factors (exotoxin A, chitin-binding protein, serine protease, and hemolysin) have been characterized as lysine-acetylated proteins (Ouidir et al., 2015). These data suggest that acetylation may play a regulatory role in the virulence of these bacteria.

### 6.2 Biofilm formation

Biofilms are bacterial multicellular communities composed of polysaccharides and proteins in which individual cells stick together and are encapsulated in a beneficial and protective environment for the microbial community (Joo and Otto, 2012). Biofilm formation is often triggered in response to environmental stresses, representing an important adaptation strategy. However, when the associated microorganisms are pathogenic, this ability becomes a significant virulence factor, protecting the bacteria from host immune responses and antibiotics (Donlan, 2000; Høiby et al., 2011).

Dental caries is a disease that occurs due to a combination of factors, such as diet, poor dental cleaning, and biofilm formation. Cariogenic and commensal microorganisms form biofilm on tooth surfaces. The factor lays the foundation for the pathogenesis of dental caries, as they provide a platform for pathogenic bacteria to colonize and accumulate (Ma et al., 2021). Among the more than 700 different bacterial species identified in dental biofilm, *Streptococcus mutans* is considered the principal etiologic agent of dental caries (Kang et al., 2011), with essential virulence factors, such as acidogenicity, aciduricity, and biofilm formation (Daboor et al., 2015; Ma et al., 2021).

The extracellular biofilm matrix is mainly managed by three kinds of glucosyltransferases (Gtfs), which synthesize extracellular polysaccharides (EPS) from sucrose and promote the adhesion of *S. mutans* to tooth surfaces (Selwitz et al., 2007). Compared with *S. mutans* in planktonic conditions, the quantitative proteome analysis revealed that the lysine acetylation levels of three Gtfs (glucosyltransferase-SI, glucosyltransferase-I, and glucosyltransferase-S) decreased in *S. mutans* in biofilm conditions (Lei et al., 2021). To determine the role of this modification, Ma and coworkers (2011) induced the overexpression of 15 genes whose products are annotated as GNATs in *S. mutans*. Only one GNATs, ActG, could enzymatically acetylate two Gtfs, reducing their enzymatic activity and water-insoluble EPS production, biofilm formation, and caries formation in a rat caries model (Ma et al., 2021). The results suggest acetylation could function as a switch-off for regulating glucosyltransferase activities (Lei et al., 2021).

In a study performed to characterize the acetylome of *M. tuberculosis*, deleting the sirtuin-like gene (rv1151c) results in the hyperacetylation of different proteins. Particularly, reversible acetylation of several fatty-acid-CoA ligases can modulate their activity. The acetylation inhibits their activity and results in a deficient biofilm formation, possibly due to the inhibition of fatty acid metabolic enzymes (Liu et al., 2014). In *Neisseria gonorrhoeae*, the AcP accumulation probably produces a nonenzymatic acetylation of the essential biofilm regulatory proteins, which leads to a marked defect in the maintenance of the biofilm structure over time. However, further analysis is required to determine which essential biofilm regulatory proteins are acetylated in *N. gonorrhoeae* (Post et al., 2017).

On the contrary, in *Yersinia pestis*, the acetylation of Lys 73 of a transcriptional regulator for the MarR-family (SlyA protein), which can regulate the expression of many genes associated with virulence and biofilm formation, inhibits its binding to the promoter of target genes and significantly enhances biofilm formation (Tan et al., 2023). In *Aeromonas hydrophila*, the S-ribosyl homocysteine lyase (LuxS) involved in the production of the quorum-sensing (QS) auto-inducer 1 (AI-1) type signal molecule, which plays an important role in biofilm formation, is acetylated at K165, and, interestingly, this site is also succinylated. Acetylation of K165 inhibits enzymatic activity, while succinylation has the opposite effect (Sun et al., 2019; Li et al., 2020a). In a previous study, the *luxS* isogenic mutant of *A. hydrophila* exhibited enhanced biofilm production and virulence in the septicemic mouse model. Therefore, the acetylation of this protein may enhance biofilm formation and virulence (Kozlova et al., 2008; Sun et al., 2019).

## 7 Acetylation regulates the response to different types of stress

Bacteria can colonize different environments since they respond quickly to fluctuating environmental stresses, including changes in temperature, pH, osmolarity, radiation, and the concentration of nutrients and toxins (Aertsen and Michiels, 2004). To ensure their survival from these adversities, bacteria coordinate various mechanisms, such as changes in the expression of the genes for combating harmful stresses, DNA repair mechanisms, elimination of molecules that cause damage (for example, reactive oxygen species), post-translational modification systems, among others (Hecker and Völker, 2001; Aertsen and Michiels, 2004).

For foodborne pathogens, the ability to survive in an extremely acid stomach environment is necessary to invade the intestinal epithelium further. Various regulatory mechanisms can be used to sense and respond to the acidic environment. *S*. Typhimurium can respond to acid stress by a reversible protein acetylation system. The *S.* Typhimurium transcriptome analysis under acid stress showed that the acetylation level may decrease through the regulation of *pat* transcription and the NAD+/NADH ratio of the cell in response to the acid signal. Furthermore, the ability to tolerate acidic conditions in the *pat* or *cobB* mutant compared to the WT strain showed that the Δ*cobB* mutant has a significantly lower survival rate than the other strains, both in the log and stationary phases (Ren et al., 2015). For other pathogens, acetylation is involved in response to oxygen limitation stress. In *Mycobacterium tuberculosis* (*Mtb*), hypoxia induces the deacetylation at K182 of DosR (dormancy survival regulon). This regulon may be a critical factor in latency adaptation, abolishing the affinity of DosR for DNA *in vitro* and further altering the transcription of DosR-regulated genes, leading to the rapid response of *Mtb* to hypoxia (Bi et al., 2018; Yang et al., 2018).

Lysine acetylation is also involved in oxidative stress protection. When reactive oxygen species exceed the antioxidant and reducing capacities within the cell, it causes damage to biomolecules such as proteins, lipids, and nucleotides (Cabiscol et al., 2000). Sodium hypochlorite (NaOCl) in solution forms hypochlorous acid (HOCl), which is a portent oxidant that causes irreversible protein aggregation by disrupting their structure (McMillan et al., 2018). The exposure to hypochlorite increases the abundance of lysine-acetylated peptides in the *H. volcanii* and Δ*sir2* mutant strains. The protein enrichment of the proteins showed significant enrichment of DNA topological change networks, AcCoA biosynthesis, and enzymes involved in translation. In the Δ*sir2* mutant, a dramatic downregulation of sulfur metabolism, cobalamin synthesis, division septum assembly, and DNA topological change proteins occurred after hypochlorite exposure (Couto-Rodríguez et al., 2023). For *E. coli*, protein lysine acetylation allows the bacterium to reach higher cell densities and become more resistant to oxidative stress. To survive this stress, some two-component system proteins may need to be acetylated for catalase activity. Interestingly, this study demonstrated that other genes are repressed by deacetylation, including genes for heat shock, osmotic stress, acid resistance, cold shock, carbon starvation, and general stress, indicating that protein acetylation is involved in various bacterial stress response systems (Ma and Wood, 2011).

Under other types of stress, it has been observed that acetylated proteins are related to metabolic enzymes, critical proteins involved in DNA damage repair and cellular processes such as translation, ribosomal structure and biogenesis, amino acid transport and metabolism, and energy conservation and conversion (Zhang et al., 2022; Xiao et al., 2023; Yao et al., 2023). These studies show a global view of the response to different types of stress and the PTM of lysine acetylation.

## 8 Lysine acetylation is involved in bacterial translation

Protein synthesis (translation) is carried out by a translational machinery composed of ribosomes, translation factors, and aminoacyl-tRNA synthetases. Ribosomes are involved in a highly dynamic and complex process of translating mRNA into proteins. They have a basic structure highly conserved in all cellular organisms, composed of two major subunits: the large ribosomal subunit (LSU) and the small ribosomal subunit (SSU). Each subunit is constituted of ribosomal RNAs (rRNAs) and ribosomal proteins (r-proteins) (Selmer et al., 2006; Ban et al., 2014; Ni et al., 2023). For example, in *E. coli*, the small ribosomal subunit comprises 16S ribosomal RNA and 24 ribosomal proteins. The large ribosomal subunit comprises a 23S ribosomal RNA and 34 ribosomal proteins (Jeffares et al., 1998).

Although different PTMs have been identified in a growing number of r-proteins and rRNAs, which regulated the fidelity of protein synthesis, including methylation, phosphorylation, and ubiquitylation, among others, the physiological role of translation-related protein acetylation, remains poorly studied (Martin et al., 2011; Sung et al., 2016; Natchiar et al., 2017). Mass spectrometry analysis of different bacterial proteomes has identified many acetylation sites on proteins. The functional annotation showed that some of the acetylated proteins are involved in translation and transcription (Weinert et al., 2013; Nakayasu et al., 2017; Christensen et al., 2019b; Marakasova et al., 2020; Liu et al., 2023).

Recently, it was found that AcP significantly reduced the relative translation rate, and deacetylation partially restored the translation activity. Using the cell-free translation system of *E. coli*, where the yellow fluorescent protein (YFP) is used as a signal output of translation activity, the YFP synthesis rate was examined after the *E. coli* extracts were incubated with AcP at different times. The analysis of the extracts by western blot to detect the acetylation level and the YFP synthesis rates of translation machinery showed a time-dependent acetylation pattern of the translation machinery in the presence of AcP and inhibition of translation by reducing the rate of elongation, while deacetylation partially restored it. Both results implied acetylation inhibits protein synthesis *in vitro* (Zhang et al., 2020a).

Sixteen acetylated sites on 12 proteins were identified by mass spectrometry. In particular, the acetylation of K411 and K464 in domains D5 and D6 of the ribosomal protein S1 reduced the chaperone activity of the protein, suggesting that acetylation may affect S1-mediated recruitment of mRNAs into the ribosome (Zhang et al., 2020a).

However, other studies showed that acetylation plays a more complex role than translational inhibition. Feid and coworkers (2022) reported that adding AcP and AcCoA inhibits translation but not transcription. The elongation rates using a LacZ induction assay were measured to confirm this observation. The results indicated that acetylation does not significantly change the elongation rate but increases the proportions of dissociated 30S and 50S ribosomes. In addition, when the 30S and 50S ribosomal subunits are disassociated, the ribosomal proteins are more acetylated than when the 70S complex is fully assembled. The authors concluded that lysine acetylation interferes with the association of ribosomal subunits, inhibiting translation (Feid et al., 2022).

The same observation was made by analyzing the acetylation of r-proteins of *Salmonella enterica* serovar Typhimurium (*S*. Typhimurium). By mass spectrometry, 289 AcK sites were identified in 52 of 54 r-proteins; of these, the small subunit protein S1 contained 19 Kac sites. Most of the acetylated lysine residues of r-proteins were found to be regulated by both the acetyltransferase Pat and the metabolic intermediate AcP. Also, it was found that acetylation plays a critical role in the assembly of the mature 70S ribosome complex by modulating r-proteins binding to rRNA and that appropriate acetylation is necessary for the interaction between elongation factors and polysomes, as well as regulating ribosome translation efficiency and fidelity (Ni et al., 2023). These data show that ribosome acetylation is crucial for their correct assembly and function.

## 9 Acetylation in gut microbiota

The human gut microbiota is a complex ecosystem composed of bacteria, archaea, viruses, and eukarya. The microbiota interacts with one another and the host in a mutually beneficial relationship, significantly impacting human health and physiology (Clemente et al., 2012). Compositional and functional alterations of the gut microbiome result in dysbiosis, which has been associated with different diseases, including obesity, diabetes, Crohn’s disease (CD), cancer, and cardiovascular diseases (Myers and Hawrelak, 2004; Clemente et al., 2012). Several studies have been carried out to determine which factors (diet, age, pregnancy, antibiotic use, among others) modulate the composition and functionality of the microbiota and how this can induce dysbiosis (Myers and Hawrelak, 2004; Brown et al., 2012; O’Toole and Jeffery, 2015; Edwards et al., 2017; Ramos and Martín, 2021), little is known about whether PTMs modifications have a regulatory effect on enzymatic activities of the microbiota and its impact.

To establish a methodology that allows studying protein acetylation in the microbiome, Zhang et al. (2020) found an experimental and bioinformatic workflow for its characterization. The proteolytic peptides were generated and aliquoted from human fecal samples for metaproteomics and acetylomics analysis. An enrichment process was carried out using a seven-plex anti-Kac peptide antibody cocktail for the lysine acetylome analysis. The metaproteomics/lysine acetylomics data analysis showed that a higher number of acetylated peptides were identified with the enrichment step. In this sample, 31,821 acetylated sites (35,200 acetylated peptides) were identified, of which 31,307 were from microbes, and 497 were of human origin. A deeper analysis revealed that protein acetylation level is much higher in Prokaryotes than human proteins in the microbiome samples and that lysine acetylation is widely distributed in gut microbial metabolic pathways, including anaerobic fermentation to generate short-chain fatty acids.

The methodology was applied to the analyses of the microbiomes of patients with CD. A total of 52 hosts and 136 microbial acetylated sites differentially abundant in disease versus controls were identified. Also, the authors demonstrated that the lysine acetylation levels in gut microbial metabolic pathways to produce short-chain fatty acids (SCFAs) were down-regulated in CD patients (Zhang et al., 2020b).

Considering that a significant challenge in metaproteomics studies is to control the discovery rate, the same group reanalyzed the previous Kac data set using an open search workflow. The open search (extensive precursor ion tolerant search) approach determines the identity of unassigned peptides using an ultra-tolerant Sequest database search that allows peptide matching even with modifications of unknown masses up to ± 500 Da (Chick et al., 2015). With this approach, they identified 57,406 Kac peptides from six microbiome samples; this is 1.6 times more. The taxonomy analysis showed that most lysine-acylated peptides were from the Firmicutes and Bacteroidetes phylum (Zhang et al., 2021). Interestingly, the study of untreated rheumatoid arthritis patients’ feces showed that lysine acetylation was downregulated and significantly positively correlated with the low relative abundance of *Fusicatenibacter* (a genus of Firmicutes) (Yu et al., 2022).

The results demonstrate that new methodologies for studying PTMs in complex samples such as the microbiome are necessary. Furthermore, these may provide more information about how PTMs regulate microbiota functions and the effect this has.

Host-microbiota interactions depend partly on metabolites produced by the microbiota that can function as receptor ligands, enzyme inhibitors, or metabolic precursors (Levy et al., 2016). Some of these metabolites can alter metabolic activities in the host through covalent modification. For example, the comparison of germ-free mice (GF) vs. colonized adult mice (CONV-D) showed that in both the colon and the liver samples, there is an increase in lysine ϵ-acetylation on CONV-D vs. GF. In addition, it was demonstrated that colonization also affected the acetylation of lysines in metabolic enzymes expressed in the liver, including the glycolytic enzyme phosphoglycerate mutase and the melanin-biosynthetic enzyme dopachrome decarboxylase. While in the colon, the most dramatically elevated site of lysine ϵ-acetylation was found in α-1-antitrypsin (Simon et al., 2012).

## 10 Methods for studying protein acetylation

To study the biological role of protein acetylation, appropriate methods must be applied to identify and quantify acetylated proteins and assign acetylation sites. Different techniques can be used in the same study, depending on the aim of the investigation, sample complexity, and type (**Table 3**).

**Table 3:** Acetylome studies in prokaryote organisms.

Organisms	Method	Acetylated site/proteins	Functional annotation			KEGG pathway	Reference
			Biological process	Molecular function	Subcellular localization		
Relative quantification method							
*E. coli*	nano-HPLC/MS/MS	125/85	Carbon metabolism, protein synthesis, and amino acid			No reported	Yu et al., 2008
*Streptococcus pneumoniae*	LC-MS/MS analysis	653/392	Cellular amide metabolic process, gene expression, and macromolecule biosynthetic process	Structural constituent of ribosome, ion binding, organic cyclic compound binding, and ligase activity	Intracellular, non-membrane-bound organelle ribonucleoprotein complexes and intracellular organelles		Liu et al., 2018
*Shewanella baltica*		2929/1103	Metabolic process, cellular process, and single-organism process	Catalytic activity and binding proteins	Cytoplasmic, periplasmic, inner membrane, outer membrane, and extracellular		Wang et al., 2019
*Brenneria nigrifluens*		1866/737	Cellular and metabolic processes	Catalytic enzymes and binding proteins	Cytoplasm, outer membrane, periplasm, inner membrane and extracellular space		Li et al., 2020b
*Campylobacter jejuni*		5567/6658	Translation and metabolic biological processes.	Biosynthesis of secondary metabolites and amino acids	Cytoplasmic, periplasmic and ribosomal proteins		Dale et al., 2023
*Vibrio alginolyticus* under bile salt stress		1,315 peptides and 689 proteins	Regulation of small molecule, organonitrogen compound process, organic substance process, single-organism metabolic process, cellular and metabolic process, and single-organism process	rRNA binding, ligase activity, RNA binding, small molecule binding, ion binding, binding, and catalytic activity	Cytoplasm, cell, intracellular, intracellular part	Metabolic pathways, biosynthesis of antibiotics, ribosome, purine metabolism, carbon metabolism, beta-Lactam resistance, and RNA degradation	Xiao et al., 2023
*Bacillus subtilis*	Nano-HPLC-MS/MS analysis	2536/866	Nucleotide-binding, RNA binding, ATP binding, and GTP binding	No reported	No reported	Glycolysis/gluconeogenesis, pyruvate metabolism, citrate cycle (TCA cycle), amino acid biosynthesis, glycine, serine, cysteine, and methionine metabolism.	Zhang et al., 2023

Quantification method							
Organisms	Labeling technique/Mass spectrometry method	Acetylated site/proteins	Functional annotation			KEGG pathway	Reference
			Biological process	Molecular function	Subcellular localization		
*Bacillus subtilis*	Label-free/Immunoaffinity enrichment/LC−MS/MS	2088/771	No reported	No reported	No reported	Translation, including ribosomal proteins and elongation factors, and enzymes involved in intermediary metabolism	Carabetta et al., 2016
*Bacillus nematocida* B16	TMT/LC-MS/MS analysis	411/288	Metabolic processes, translation, macromolecule biosynthesis, and tRNA aminoacylation	Information storage and processing, cellular processes and signaling, and metabolism	Cytoplasmic component and ribosomal unit	Acetylated proteins were involved in many pathways (pyruvate metabolism, glycolysis, the citrate cycle pathway, and purine metabolism)	Sun et al., 2018
*E. coli* strains	Label-free/LC-MS/MS analysis	818/434	Ribosomal protein subunits: Amino acid-tRNA ligases Elongation factors Initiation factors	No reported	No reported	Glycolytic pathways	Christensen et al., 2018
*Clostridium* *acetobutylicum*	Stable Isotope Dimethyl Labeling/Nano-HPLC-MS/MS	458/254	No reported			Ribosome, biosynthesis of amino acids, purine metabolism, glycolysis/gluconeogenesis, amino acid degradation, butanoate metabolism, pentose phosphate pathway, fatty acid metabolism, and pyruvate metabolism	Xu et al., 2018
*Pseudomonas aeruginosa*	Label-free/2D immunoaffinity approach coupled/nano HPLC/MS/MS	1102/522	No reported	Amino acid metabolism, translation, and central carbohydrate metabolism.		Fatty acid (FA) biosynthesis and degradation, glycolysis/Gluconeogenesis and Citric acid cycle	Gaviard et al., 2018
*Streptococcus mutans*	TMT labeling/Immunoaffinity enrichment/LC−MS/MS	973/445	Metabolic process, cellular process, binding, catalytic activity, and cell	Carbohydrate metabolism and catabolism, and glucan metabolic process	Cytoplasmic and membrane	Glycolysis/gluconeogenesis and RNA degradation	Lei et al., 2021

As a first step in the investigation, detection, and semi-quantification of the targeted acetylated protein can be made by western blot analysis using specific antibodies against acetylated proteins (Yu et al., 2008; Hirano, 2012; Diallo et al., 2019). This type of analysis gives a global view of the acetylation profiles under different conditions (temperature, growth phase, stressors, among others) and in different strains to determine the differences in acetylation levels qualitatively (Kosono et al., 2015; Schilling et al., 2015; Christensen et al., 2018; Xu et al., 2018; Dale et al., 2023). However, this approach usually presents an insensitive and non-specific signal, so it is necessary to introduce negative controls to demonstrate the specificity of the anti-acetyl lysine antibody. Furthermore, it is not possible to know the number/assignment of specific acetylation sites, and protein identification requires additional steps (Diallo et al., 2019).

Liquid chromatography coupled to mass spectrometry (LC-MS/MS) systems has become the method for sequencing and identifying thousands of proteins. Due to its sensitivity, precision, and accuracy, it is also necessary to study PTMs.

Mass spectrometry has been widely used for global acetylome analysis in several bacterial species. The data generated are commonly used to determine the number of proteins and acetylated sites and the metabolic processes that this modification regulates through the functional annotation of the acetylated proteins and identify the potential consensus motifs of the acetylated sites (**Table 3**). One of the first published acetylomes in bacteria was from *E. coli* using an anti-acetyl lysine antibody with nano-HPLC/MS/MS. This study identified 125 lysine-acetylated sites in 85 proteins involved in diverse physiological functions, including carbon metabolism, protein synthesis, and amino acid metabolism (Yu et al., 2008). In the following years, many groups conducted global screening and characterization of lysine acetylation in other organisms. In *Streptococcus pneumoniae*, 653 lysine-acetylated sites on 392 proteins were identified. The gene ontology enrichment analysis demonstrated that in the metabolic process, the major targets of lysine acetylation are involved in the cellular amide metabolic process, gene expression, and macromolecule biosynthetic process. The acetylated proteins identified mainly possess structural constituents of the ribosome, ion binding, organic cyclic compound binding, and ligase activity for molecular function. The acetylated proteins were significantly enriched in intracellular, non-membrane-bound organelle ribonucleoprotein complexes and intracellular organelles for cellular components GO enrichment analysis. The motif analysis of the identified acetylated peptides showed that all the amino acids in these motifs are hydrophilic (Liu et al., 2018). Moreover, the KEGG pathways enrichment analysis allows further insights into the functional correlation of lysine acetylation. In *V. alginolyticus*, the functional correlation of acetylation and bile salt stress through KEGG pathways enrichment analysis revealed that the largest group of acetylated proteins was involved in metabolic pathways (20.5%), biosynthesis of antibiotics (13.3%), ribosome (5.8%), purine metabolism (3.3%), carbon metabolism (2%), beta-lactam resistance (2.0%), and RNA degradation (1.7%). In general, these data indicated that the enzymes in the metabolic pathway play an important role in enabling bacteria to respond to stress (Xiao et al., 2023).

To better understand how lysine acetylation regulates protein function and if this mechanism is evolutionarily conserved, Nakayasu and coworkers investigated by LC-MS/MS whether acetylation occurs on lysine residues conserved in different bacteria. For this, the proteomic analysis of 48 phylogenetically diverse bacteria from six phyla: *Proteobacteria*, Firmicutes, Bacteroidetes, Actinobacteria, Cyanobacteria, and Fibrobacteres were analyzed. The data showed that acetylated proteins were significantly enriched in glycolysis, pyruvate metabolism, ribosomes, and the TCA cycle. Some enzymes involved in glycolysis and the TCA cycle were further examined to analyze whether these enzymes contained catalytically critical lysine residues involved in substrate or cofactor binding. The results showed that lysine residues are localized in the active site that binds to the substrate or cofactor, demonstrating that lysine acetylation occurs in evolutionarily conserved residues of the enzyme catalytic sites in multiple organisms (Nakayasu et al., 2017). Moreover, the in-silico modeling analysis adding acetylation to the enzyme structure evidence that the modification alters the charge and shape of the lysine residue by neutralizing its positive charge and increasing its size; therefore, the catalytic activity is inhibited (Nakayasu et al., 2017).

Mass spectrometry is not an inherently quantitative method because proteolytic peptides have different physiochemical properties (size, charge, hydrophobicity, and more), which produce variations in the MS/MS spectra. Therefore, for accurate quantification, it is generally required to compare each peptide between experiments (Bantscheff et al., 2007). For this reason, different labeling techniques coupled to LC-MS have been developed: stable-isotope labeling with amino acids in cell culture (SILAC), isotope-coded affinity tags (ICAT), tandem mass tag (TMT), iTRAQ, multiplex isobaric tags, and heavy peptide AQUA are some examples (Gingras et al., 2007; Lindemann et al., 2017; Zhang and Elias, 2017).

As shown in **Table 3**, using different labeling strategies, it has been possible to quantify acetylated sites and proteins robustly and precisely, which has allowed us to elucidate the role of N-acetylation in processes such as biofilm formation and pathogenesis, determine how the dynamics of acetylation during bacterial growth and if the carbon source influences the PTM rate. For the pathogenic bacterium *Bacillus nematocida* B16 (B16), an antagonistic against plant-parasitic nematodes, the lysine acetylome quantification using TMT-(Tandem Mass Tag) labeling during its interaction with worms allowed determining that the acetylation levels of 18 sites were up-regulated and those of 19 sites were down-regulated during pathogenesis. Acetylated peptides were further characterized based on the Gene Ontology annotation (COG). The analysis suggested that acetylated proteins participate in diverse biological processes, mainly in metabolism and protein synthesis. Furthermore, analysis of the acetylated proteins revealed that many identified proteins are involved in B16 infection, suggesting that lysine acetylation may play a role in regulating B16-*C. elegans* interaction (Sun et al., 2018). In *E. coli*, label-free quantification allowed to determine the substrate specificity of four new Nϵ-lysine acetyltransferases. Using an *E. coli* strain that lacked known acetylation mechanisms (YfiQ and AcP), 818 acetylation sites on 434 proteins whose acetylation increased with overexpression of at least one KAT were identified. Of the proteins identified as acetylated by the newly identified KATs, several are central metabolic proteins, and many are components of the translational machinery. These new Kats tend to acetylate enzymes that regulate the branch points of central metabolism, while AcP seems to modify many of the central metabolic enzymes. These suggest that enzymatic acetylation has evolved to regulate key flux points in metabolism specifically and that non-enzymatic acetylation is a mechanism global to respond to the carbon flux (Christensen et al., 2018).

Although relative quantification is a powerful technique that detects variations in the acetylation levels in different conditions, it is limited by the fact that the changes are relative to the total protein abundance, which can vary from one condition to another, giving misinterpretations about the physiological significance of this PTM. Analysis of acetylation stoichiometry or occupancy can allow us to identify the critical acetylation sites whose changes in abundance are important.

However, determining the acetylation stoichiometry is complex, mainly because the ionization efficiency of modified and unmodified peptides in a mass spectrometer is different and artifact-prone. For this reason, various working groups have reported methods and workflows for the precise quantification of site-specific protein acetylation occupancy, which are based on the comparison of the proportion of endogenously acetylated lysine versus chemically labeled lysine that is not endogenously acetylated (Baeza et al., 2014; Weinert et al., 2014; Gil et al., 2017; Miyagi, 2017; Weinert et al., 2017; Wei et al., 2018).

The first protocol developed for directly quantifying stoichiometry of site-specific acetylation in bacteria was based on the chemical acetylation of free lysine residues with isotopic acetic anhydride, followed by trypsin cleavage and MS analysis. The method was applied to analyze the entire proteome of *E. coli* precisely to determine the role of deacetylases, CobB, on both site-specific and global acetylation (Baeza et al., 2014). In a similar study, Weinert et al. (2017) determined the absolute acetylation stoichiometry but used a serial dilution of SILAC-labeled peptides (SDSILAC). Although the methodologies differ in the way of labeling the peptides, with both approaches, it was shown that sirtuin deacetylase deficiency affects central metabolism and leads to both site-specific and global changes in protein acetylation stoichiometry (Baeza et al., 2014; Weinert et al., 2017).

Exploring the relationship of chemical acetylation and how it affects the enzymatic activity of glycolytic proteins, it was found that possibly a maximum of 10% of non-enzymatically acetylated proteins reach a stoichiometry that could inhibit their activity and that enzymes such as GapA and GpmA are acetylated at high stoichiometry (Schastnaya et al., 2023). The authors suggest that AcP-acetylation is specific and may exert control over metabolism.

Knowing the stoichiometry of acetylation can help to establish how it changes and whether it exerts a regulatory effect or only has a constitutive function necessary for protein folding or stable interaction.

### 10.1 Genetic code expansion

The advances in mass spectrometry techniques enabled researchers to identify thousands of acetylation sites in various proteins. As mentioned throughout the review, analyzing the acetylome of multiple organisms using either stable isotope labeling workflows or label-free protocols or analyzing the acetylation through stoichiometry measurements has shown that acetylation regulates gene expression and physiological processes. However, it should be considered that lysine acetylation is highly dynamic and mostly reversible in cells. Therefore, the extraction of acetylated proteins with identical acetylation stoichiometry is difficult. Also, chemical lysine acetylation usually occurs at multiple sites simultaneously in one protein, and other modifications can compete with acetylation for the same lysine residue (Fatema and Fan, 2023). Although different methods have been developed to overcome these limitations, such as direct mutagenesis to mimic the acetylated lysine residue (substitution of lysine for glutamine), the study of acetylation remains challenging mainly because the substitution only imitates the electronic effects of the modifications but not their steric effects.

The genetic code expansion technique (GCE) has been applied to generate site-specifically acetylated proteins. Some reviews summarize the use of this technique for the analysis of different PTMs and the strategies to produce site-specifically modification (Neumann et al., 2008; Schmidt and Summerer, 2014; Krauskopf and Lang, 2020; Neumann-Staubitz et al., 2021; Chen and Tsai, 2022; Fatema and Fan, 2023). Specifically, proteins with the actual modifications at the desired positions can be generated recombinantly, facilitating biochemical studies (Chen and Tsai, 2022).

The GCE strategy to site-specifically incorporate Nϵ-thioacetyl-l-lysine (TAcK) as an analog of Nϵ-acetyl-l-lysine (AcK) into green fluorescent protein and malate dehydrogenase (MDH) in *Escherichia coli*, showed that TAcK could serve as an ideal functional mimic for AcK. The replacement of lysine residue 140 in MDH showed that the MDH-140TAcK variant could increase the enzyme activity by 3-fold, like that of the MDH-140AcK variant (Venkat et al., 2017b). Another enzyme studied with this approach is the aconitase of *E. coli*. Aconitase catalyzes the second reaction of the tricarboxylic acid cycle, the reversible conversion of citrate and isocitrate. With GCE 14, site-specifically acetylated aconitase variants were produced, and their enzymatic analysis and kinetic analyses showed that acetylation of AcnA K684 decreased the enzyme activity. In contrast, acetylation of AcnB K567 increased the enzyme activity. Moreover, *in vitro* acetylation and deacetylation assays were performed, and the results showed that the enzyme could be acetylated by acetyl-phosphate chemically and be deacetylated by the CobB deacetylase at most lysine sites (Araujo et al., 2022).

GCE has been used to study the effect of coexisting multiple distinct PTMs in one protein. Malate dehydrogenase was used to validate the method, and phosphorylation and acetylation were studied simultaneously in *E. coli*. The simultaneous incorporation of phosphoserine (Sep) and acetyl lysine (AcK) was confirmed by both Western blotting, and then the enzyme activities of wild-type, AcK only containing, Sep-only containing, and AcK/Sep-containing MDHs were measured. Lysine acetylation at position 140 increased the enzyme activity by 3.5-fold, while serine phosphorylation at position 280 decreased the enzyme activity to only 30% of that of the wild-type enzyme. Interestingly, the combination of 140-AcK and 280-Sep had a similar activity with the wild-type enzyme, indicating that two PTMs work separately to affect MDH activity (Venkat et al., 2018b).

These studies demonstrated practical applications of genetic code expansion in acetylation studies.

## 11 Remarks

In recent years, the field of research on acetylation in bacteria has increased considerably, demonstrating its relevance in various cellular processes. Protein acetylation is a highly dynamic modification that occurs on short time scales (minutes to hours), where the acetylation levels are dictated by two closely related mechanisms: the addition and removal of the acyl group (Baeza et al., 2020). This property allows the cell to respond quickly to different environmental changes by modifying the functionality of the protein to adjust metabolic processes.

The development of protein labeling and enrichment methods and technological advances in mass spectrometry for identifying and quantifying acetylated sites and proteins have allowed the study of lysine acetylation in various organisms under different conditions. Thus, it has been shown that acetyl-lysines must result from one of the recognized acetylation mechanisms, whether enzymatic or non-enzymatic. Furthermore, it has been identified that the target acetylation proteins participate in various cellular and metabolic processes, highlighting the acetylation of conserved lysines in central metabolism proteins. However, for >99% of proteins, the biological relevance of this modification remains to be determined.

Another critical aspect yet to be investigated profoundly is the determination of the acetylation stoichiometry. Quantification of fold changes only sometimes has crucial biological significance. For example, if acetylation has functional consequences, such as loss of function, the modification should have a high stoichiometry. In comparison, a low percentage of acetylation may suggest a modification related to protein folding or the formation of a stable interaction (Carabetta et al., 2016). So far, the techniques available for determining the stoichiometry have revealed that most acetylated proteins occur at low stoichiometry (0–10%), and in approximately 4% of acetylated sites, a >20% stoichiometry is observed (Baeza et al., 2014; Weinert et al., 2017). This indicates that only a tiny fraction of the acetylome is of physiological importance. Also, it is essential to consider that proteomic analysis is based on experiments under a particular condition and in isolated organisms, so it would be convenient to consider performing a comparative analysis under different conditions (temperature, carbon sources, among others) and their behavior in co-culture or symbiosis for a correct interpretation of the data.

Several functional monitoring studies are still being carried out using *in vitro* assays. Although they allow us to know the effect of acetylation on the protein, they only sometimes reflect the natural consequence of this post-translational regulation process. Therefore, it is necessary to generate hybrid approaches that help us identify and characterize not only the modified proteins but also which metabolic pathways are involved and how this impacts the cell’s metabolism. Such approaches can integrate the analysis of proteomic, transcriptomic, and metabolomics data sets.

Incorporating synthetic biology can expand our understanding of how lysine acetylation regulates protein function. In this regard, genetic code expansion (GCE) has become essential for studying biological processes such as post-translational modifications. Rather than adding acetyl groups after protein translation, this approach relies on heterologous pairs of aminoacyl-tRNA-synthetases (aaRS) and its cognate tRNA from *Methanosarcinaceae* species that enable the co-translational incorporation of Nϵ-acetyllysine (AcK) in response to stop codon (Schmidt and Summerer, 2014). This approach has been used for some groups to evaluate the role of lysine acetylation using recombinantly expressed proteins, finding that the enzymatic activity of several proteins is positively or negatively regulated. Once again, we believe this type of study should be applied to studies of bacteria *in vivo*.

Non-enzymatic acetylation is an inevitable consequence of the cell’s metabolic states. At high concentrations of AcP this mechanism is favored. However, it should be considered that this molecule can also be used as an acyl group donor for acetyltransferases catalyzing protein acetylation. Therefore, it cannot be ruled out that these mechanisms coexist. The roles of global acetylation and other acyl donors (succinyl-CoA, propionyl-CoA, and malonyl-CoA) for enzymatic or non-enzymatic protein acylation remain to be elucidated. Finally, it is essential to understand how lysine acetylation interferes with and cross-links with other post-translational modifications.

## Author contributions

JR: Writing – review & editing, Writing – original draft. SE-G: Writing – review & editing, Writing – original draft.
